# Music Therapy with Preterm Infants and Their Families after Hospital Discharge: An Integrative Review

**DOI:** 10.3390/ijerph21081018

**Published:** 2024-08-02

**Authors:** Verena Clemencic-Jones, Suza Trajkovski, Allison Fuller, Karen Mattock, Virginia Stulz

**Affiliations:** 1 School of Nursing and Midwifery, Western Sydney University, Locked Bag 1797, Penrith, NSW 2751, Australia; s.trajkovski@westernsydney.edu.au; 2 School of Humanities and Communication Arts, Western Sydney University, Locked Bag 1797, Penrith, NSW 2751, Australia; a.fuller@westernsydney.edu.au; 3 School of Psychology, Western Sydney University, Locked Bag 1797, Penrith, NSW 2751, Australia; k.mattock@westernsydney.edu.au; 4 School of Nursing and Midwifery, University of Canberra, Bruce, ACT 2617, Australia; virginia.stulz@canberra.edu.au

**Keywords:** preterm infants, family, music therapy, after hospital discharge, integrative review

## Abstract

After discharge from a neonatal unit, families of preterm infants may require therapeutic support to address challenges related to their infant/s’ development, changed family circumstances, and/or parent wellbeing. This integrative review (IR) sought to examine the impact of music therapy on preterm infants and their families post-hospital discharge. A systematic search encompassing seven databases resulted in 83 citations, with six studies initially meeting the inclusion criteria. A further six studies were evaluated and selected upon their publication during the review process. Each study was assessed using the Mixed Methods Appraisal Tool (MMAT), followed by the identification of major themes and sub-themes. Our results suggest that music therapy contributed to creating supportive physical and metaphorical environments for preterm infants and their families, in which they could acquire essential skills, tools, and resources for fostering communication and connection with one another. Preterm infants and toddlers may have also enhanced their developmental skills through music therapy sessions post-discharge. Further investigation into the impact of music therapy on preterm infants and their caregivers at different timepoints after hospital discharge is recommended, as well as a comparison of individual and group music therapy outcomes on infant development and parent health. Future research should include a broader spectrum of family members, along with caregivers from diverse family structures and gender identities, reflecting practices already established in some clinical settings.

## 1. Introduction

Globally, an estimated one in eleven pregnancies results in a preterm birth (when an infant is born at less than 37 completed weeks of gestation). Of these babies, approximately 85% are moderate to late preterm (32–37 weeks), another 10% are very premature (28–32 weeks), and the remaining 5% are born extremely premature (less than 28 weeks gestational age (GA) [1,2]. Due to technological advancements and improved hospital care, the overall survival rates for preterm babies have risen [3]; however, epidemiological data indicate many preterm infants are at increased risk of neurodevelopmental delays compared to those born at term or equivalent [4,5]. Additionally, parents of preterm infants are susceptible to a combination of physical, emotional, and behavioural distress resulting from their infants’ hospitalisation in a neonatal intensive care unit (NICU) [6,7]. Following hospital discharge, some caregivers may therefore experience challenges as they come to terms with their infants’ ongoing health requirements and the resulting impact on family life, and their own emotional and physical wellbeing [8].

In many countries, interdisciplinary follow-up teams offer a crucial service by monitoring the neurodevelopment of infants born premature and providing referrals to health care clinicians delivering early therapies and parenting programs targeted at this population [9,10]. A Cochrane review of early developmental interventions after hospital discharge determined that both infant neurodevelopment and parent outcomes were enhanced by parental presence and participation in their infant/s’ therapies [11]. Similarly, a recent program developing parental responsiveness strategies increased infant developmental outcomes and promoted positive parent mental health [12,13]. However, therapy focusing solely on infant health post-hospital discharge appeared to be less effective for preterm babies at high risk of poor neurodevelopmental outcomes than those in which parental wellbeing was also addressed [14,15].

Music therapy (MT), an evidence-based allied health profession [16], is offered as a therapeutic modality in numerous neonatal units worldwide [17]. It is supported by a substantial body of research [18,19,20], including Haslbeck’s integrative review (IR) from 2012, which found researchers had primarily investigated the effects of receptive music on hospitalised preterm infants and suggested future NICU-based studies comprise: (a) live music therapy interventions, (b) qualitative and quantitative research methods, and (c) parental perspectives [21]. From this timepoint, research has demonstrably progressed in this direction. A multisite NICU MT study found parent parent–infant co-regulation when a parent or music therapist sang a ‘song of kin’ (parent-selected lullaby/culturally familiar song) to a preterm infant, with this song intended to accompany the child from hospital to the home environment and through early childhood [22]. Correspondingly, a mixed methods investigation demonstrated that a music therapist-guided intervention encouraging daily maternal singing/humming during NICU-based kangaroo care resulted in increased maternal wellbeing and mother–infant bonding for participants compared to a control group. Furthermore, all mothers in the singing intervention group (*n* = 21) continued singing to their babies at home post-discharge [23].

Recommendations to provide MT beyond the neonatal environment have gained support from NICU parents [24] and contemporary NICU MT researchers. Haslbeck et al. [25] proposed MT commence in the NICU followed by group MT sessions and individual home visits post-hospital discharge, with Kostilainen et al. [26] highlighting a similar need for research in this area, and Menke et al. [27] suggesting family-centred outpatient MT be implemented after hospital discharge. However, despite various studies advocating the benefits of family-centred, early intervention (EI) programs for premature infants post-discharge [28,29,30], research into MT clinical practice with preterm infants and their families after hospital discharge remains limited. Surprisingly, a systematic search of the NICU MT literature revealed multiple descriptions of—and recommendations for—MT in the post-discharge phase and beyond [31,32]. For instance, Dr Monika Nöcker-Ribaupierre, a pioneering NICU MT clinician researcher, began studying the mother’s singing voice in 1987. She defined it as a primary source of bonding between a mother and her preterm infant/s, a sonic bridge linking a baby’s life in utero to their NICU experience, followed by transition to the home environment post-hospital discharge [33]. The music therapist’s role in this context was to protect and nurture this connection [34]. Likewise, Dr Jayne Standley, another clinician researcher at the forefront of NICU MT research, has consistently advocated for the use of MT in EI with former preterm infants to promote mother–infant bonding [35], social interaction, and developmental milestones [36].

Post-NICU discharge MT clinical protocols were also mentioned in a review of NICU-based MT research [37], while a recently published textbook described a home-based individual MT program conducted in the first six months after hospital discharge [38]. A case study explored how former preterm two-year-old sisters and their developmentally delayed triplet sibling developed the ability to share, play, and creatively connect during a short-term MT program [39]. Grey literature included a conference abstract outlining the protocol for a randomised controlled trial of post-discharge group MT and waterplay sessions [40], as well as webpages outlining the *Sing with Me program* [41] and the *Sounds of Love in Early Childhood* [42] MT class for former preterm infants and children from the general community in hospital outpatient settings.

In 2008, NICU MT clinician researchers Dr Deanna Hanson-Abromeit, Dr Helen Shoemark, and Dr Joanne Loewy recommended MT as a necessary clinical service in NICU outpatient follow-up clinics [43]. Nonetheless, between 2009 and 2018, we only found three studies on MT with preterm infants and their families after hospital discharge [44,45,46]. Following this, there was an influx of MT research articles—commencing with a clinical protocol for the Longitudinal Study of music Therapy’s Effectiveness for Premature infants and their caregivers (LongSTEP) project [47]. This international multisite randomised controlled trial aimed primarily to measure changes in mother–infant bonding after an infant-directed parental singing intervention in the NICU and/or post-discharge. Further articles associated with LongSTEP encompassed those related to the introduction of MT in the NICU and after hospital discharge in Poland [48,49], and one detailing LongSTEP’s theoretical framework and intervention protocol [50]. These articles have much to contribute to the development of MT with preterm infants and their families after hospital discharge; however, they lie beyond the bounds of this IR.

As evidenced above, the publication of post-discharge MT research has significantly increased in the past five years, although combined findings from diverse programs have not been thoroughly appraised. Given that research into the impact of MT post-NICU discharge could potentially influence and guide future clinical practice and research, we seek to bridge this gap in the knowledge with an IR.

Our primary review questions were: how did MT affect preterm infants and their families after hospital discharge, and which interventions and approaches were used to promote infant development? Hence, the aim of our IR was to determine the impact of MT on preterm infants and their families following hospital discharge. This was achieved by identifying relevant research studies, comparing and contrasting clinical methods, synthesising emerging themes, and identifying gaps in the existing knowledge.

## 2. Materials and Methods

### 2.1. Methodology

We chose the IR method as it employs a rigorous, systematic, and transparent approach that incorporates varied methodologies (quantitative, qualitative, mixed method) and perspectives, resulting in more comprehensive insights into specific phenomena [51]. This review followed a six-step process, or methodological framework, based on the protocol introduced by Cooper [52] and adapted by various researchers, including Whittemore and Knafl [53], Russell [54], Torraco [55,56], and deSouza [57]. It comprised: 1. formulation of a research purpose and/or review question, 2. systematic search and selection of the literature, 3. literature quality appraisal, 4. literature analysis and synthesis, 5. discussion and conclusion, and 6. dissemination of findings [51].

### 2.2. Search Terms and Strategies

Two researchers (VCJ and VS) identified search terms via brainstorming after a prior reading of research-related literature (see Table 1). Both controlled and natural language terms were used, with Boolean Operators (AND, OR) merging terms within—and between—individual search strategies. The truncation of certain keywords enhanced the potential for increased search results, whereas phrase-searching enabled exact searching of entire concepts [58]. 

We accessed multiple online databases—APA PsycINFO, CINAHL Full text, Ovid MEDLINE, ProQuest Central, SAGE Publishers, Scopus and Web of Science—commencing our initial search in November 2021, with monthly checking for relevant updates until December 2023. Reference lists were also hand searched to identify further potential studies. These searches generated 12 articles [44,45,46,59,60,61,62,63,64,65,66,67], which were imported into Mendeley Reference Manager (Desktop, Version 1.19.8).

### 2.3. Inclusion and Exclusion Criteria

This IR encompassed any studies reporting on MT with preterm infants and their families during the first two years after preterm birth. Primary research studies with quantitative, qualitative, and mixed methodologies were included, not only to source as many relevant articles as possible but also to obtain multiple perspectives on this subject area. Secondary research studies, research protocols, and clinical recommendations were excluded.

Due to the initial paucity of available articles in the recommended 10-year time frame [68], expanded time limiters were placed on publishing dates, with reviewers considering any studies published after 1990. This resulted in the retrieval of a landmark publication in which the concept of ‘early intervention’ MT for preterm infants post-hospital discharge was first mentioned [69]. While not meeting inclusion criteria for this IR, the article nevertheless identified a historic timepoint (1998) at which MT researchers writing in the English language began contemplating the development of MT with preterm infants beyond the NICU environment. Extending the time frame resulted in the extraction of two quantitative studies published in 2009 [44,45], which, after appraisal, were included in the review.

This review only incorporated articles written in English as it is considered a global research language [70], and the majority of preterm infant-related MT research is in English. We included studies in which university-trained music therapists used ‘live’ therapeutic music methods post-NICU discharge. Excluded were studies categorised as MT in which recorded music was used without the input of a trained music therapist; a phenomenon commonly encountered in research related to therapeutic uses of music in the NICU [71,72,73]. Peer review was the final inclusion requirement, as this is the scholarly gold standard for academic publications and minimises the risk of professional and publication bias [74].

### 2.4. Search Outcome

**Figure 1:**
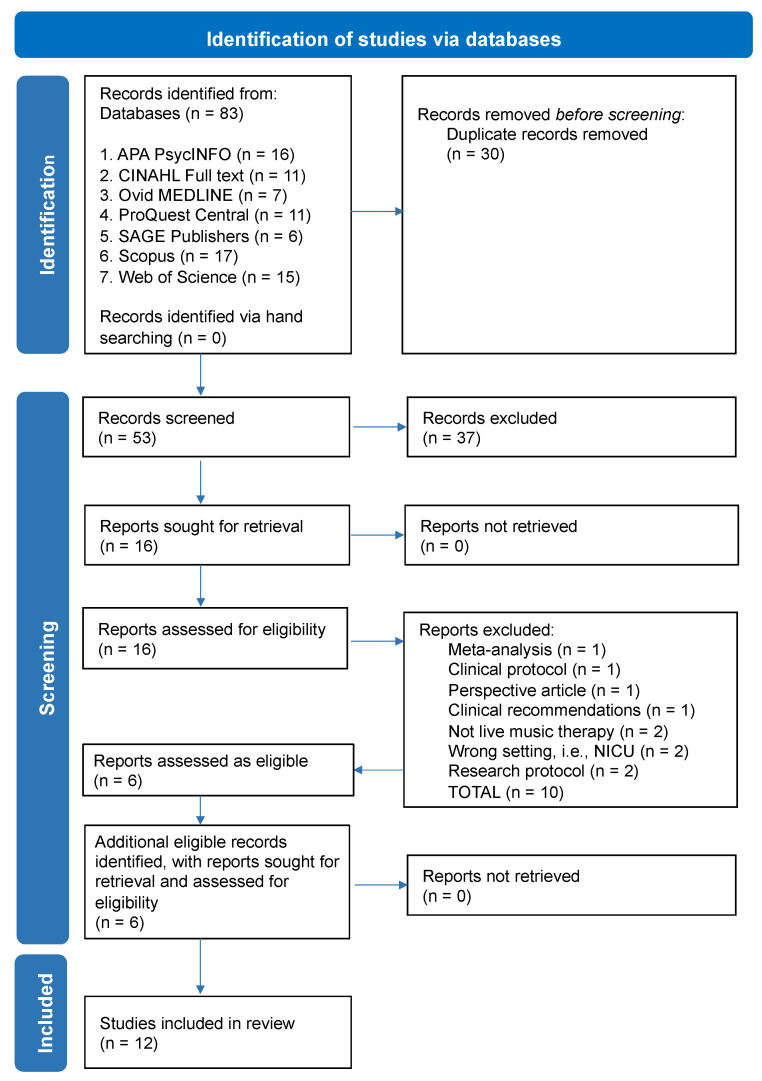
PRISMA flow diagram—search strategy for MT post NICU discharge. Adapted from the 2020 PRISMA statement [75].

We documented the search strategy through a PRISMA flow diagram, which clearly guides reviewers to systematically detect, choose, evaluate, and synthesise research studies [75] and is recommended when conducting an IR [51]. The initial database search yielded 83 articles, with 30 records removed before screening due to duplication. Two researchers (VCJ and VS) screened the abstracts of the remaining 53 articles with a further 37 records excluded due to: the wrong setting, wrong population, or not being MT. Sixteen reports were sought for retrieval and subsequently assessed for eligibility by VCJ, VS, ST, AF, and KM. Six articles were selected for inclusion, with 10 excluded for diverse reasons (see Figure 1). A further six articles were included after reviewer screening in 2022/2023, culminating in a total of 12 studies in this integrative review.

### 2.5. Quality Assessment

The Mixed Methods Appraisal Tool (MMAT) [76] was used to appraise the quality of each research study, with VCJ and VS assessing all articles, and co-researchers ST, AF, and KM assessing four articles each. The MMAT is a suitable critical appraisal tool for literature reviews comprising diverse research methods as it has multiple research design categories (qualitative, quantitative randomised controlled trials, quantitative non-randomised, quantitative descriptive, and mixed methods), with each study rated in terms of the appropriate criteria [77]. No studies were excluded, as every article’s quality score was suitable for inclusion in this IR (see Table S1, Supplementary Materials file). Nonetheless, our interdisciplinary team of MT, nursing and midwifery, and psychology researchers engaged in robust discussion while appraising certain articles. For example, the inclusion of one paper was extensively debated, as it contained minimal information but still met all the required criteria to attain the maximum score for a quantitative non-randomised study [46]. We could not assign the LongSTEP feasibility studies a single research category in which to be appraised as they encompassed qualitative and quantitative data collection methods without being mixed methods studies [59,60]. Hence, we scored these articles using both qualitative and quantitative screening questions.

### 2.6. Data Analysis

Data were analysed using Whittemore and Knafl’s constant comparison method in which data are reduced, displayed, and compared prior to drawing conclusions and verification [53]. Initially, VCJ extracted information from each article into relevant characteristics, displaying them in a data review matrix (see Table S1, Supplementary Materials file). This table detailed the author/s, year of publication, country, study aims, participants, settings, study design, interventions/therapeutic techniques, data collection and analysis, and results/findings. This contributed to our understanding of the development of MT with preterm infants and their families after hospital discharge as a phenomenon of interest to both clinicians and the research community [78]. Certain queries arose from the data extraction process, such as whether it were feasible to: (a) omit data referring to MT in the NICU from six LongSTEP studies [59,60,64,65,66,67], or (b) separate preterm from full term infant data in studies focusing on group MT after hospital discharge [44,45,46]. In both cases, the research team decided to incorporate all data in the integrative review matrix as it would have otherwise compromised each study’s integrity, but to include primarily post-NICU discharge data in the analysis of themes, as this review’s focus was MT with preterm infants and their families after hospital discharge.

### 2.7. Data Synthesis

Although each study in this IR was influenced by unique geographic, cultural, clinical, and research contexts, we found similar themes and sub-themes emerging from our analysis. After dividing and grouping the data into different methodologies and general themes, VCJ and VS coded each journal article into segments of meaning (sub-themes) using MAXQDA 2022 software, Version 22.8.0 [79] and manual coding techniques. Patterns (similarities and differences) in the data were identified and, after discussion with the research team, individual sub-themes were merged into four major themes and 12 sub-themes, forming the structure of the Results section [55,78]:

1. Creating supportive environments, with the sub-themes:

1—a physical or metaphorical space, 2—a space for insight and self-expression, 

3—a space of calmness or engagement;

2. Developing skills, tools, and resources, with the sub-themes:

1—infant development, 2—parent development, 3—music therapist development;

3. Acknowledging challenges, with the sub-themes:

1—infant challenges, 2—parent challenges, 3—research challenges;

4. Building relationships, with the sub-themes:

1—infants and parents, 2—infants and others, 3—parents and others.

**Figure 2:**
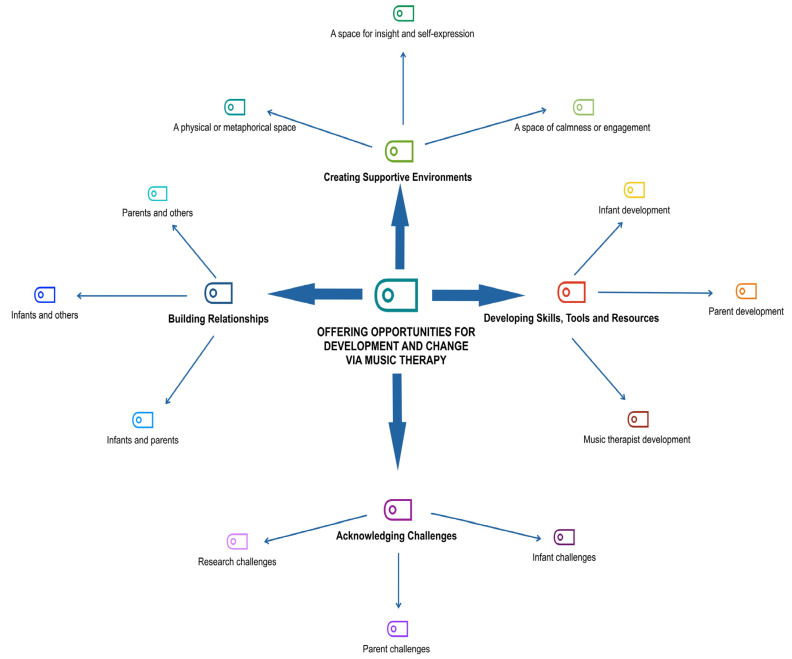
MAXQDA creative coding map of integrative review themes.

Multiple connections were encountered between, and across, the four major themes, leading to the emergence of a single overarching theme, *Offering Opportunities for Development and Change* via *Music Therapy*. A visual representation of the IR themes is presented below (Figure 2).

A MAXQDA word frequency count of the overarching theme’s vocabulary was undertaken, which found words originating from each of the four stems ‘offer*’, ‘opportunit*’, ‘develop*’ and ‘chang*’ in the majority of the reviewed studies, as well as the term ‘music therapy’ in every study. Word definitions related to the central theme and its four major themes can be found in the Supplementary Materials file—Additional Notes 1.

We also discovered frequent interwoven and intersecting commonalities while coding themes for this IR. For instance, the parental acquisition of a skill led to a growth in self-confidence, resulting in an enhanced ability to communicate and connect with their babies [61,62,63,65,67]. These overlapping relationships were evident across multiple themes and sub-themes, with certain studies using diverse terms for similar constructs with comparable outcomes, such as musical and parenting confidence [63]; communicative parental efficacy [61]; musical and parental agency [62,65,67]; and emotional [62], musical [59,60,62,65,67], parenting [60,62,67], and personal [63] resources. Table 2 presents the frequency with which each sub-theme occurs. 

## 3. Results

All 12 studies selected for this integrative review were published between 2009 and 2023 in Q 1 or 2 academic publications, ranging from the *Nordic Journal of Music Therapy* to *JAMA Network Open*. Each study was undertaken by university-trained MT researchers either supported by, or in collaboration with, medical, nursing, and allied health clinicians and researchers. The research was conducted internationally, in Australia (*n* = 1), Israel *(n* = 4), Norway (*n* = 1), Poland (*n* = 1), the USA (*n* = 3), and across multiple sites: Argentina, Colombia, Israel, Norway, and Poland (*n* = 2) [44,45,46,59,60,61,62,63,64,65,66,67].

Study aims varied from investigating the effects of structured group sessions on full term and preterm infant toddler development [44,45,46] to exploring the impact of individual MT on infants and their families [61,62,63,65]. Three studies aimed to evaluate the feasibility [59,60] and treatment fidelity [64] of the LongSTEP protocol. The main LongSTEP study sought to investigate MT’s effect on parent-infant bonding, parent mental health, and infant development [66], whereas another LongSTEP-based study proposed to examine the construct of ‘musical agency’ as a framework for understanding MT’s effect on families of preterm infants in hospital and post-discharge [67].

Quantitative sample sizes ranged from 22 to 213 participants and employed the following sampling techniques: convenience sampling [44], quasi-experimental matched (self-selected) sampling [45], case-control sampling [46], and random sampling [64,66]. Purposive sampling was used for all the remaining studies [59,60,61,62,63,65,67], with sample sizes ranging from one to seven participants. Preterm infants’ GA at birth were not mentioned in two studies [44,45], with all remaining articles either providing age ranges from 26 to 34 weeks GA [59,60,61,62,63,65,67] or a mean GA at birth [46,64,66]. Preterm infants’ ages at the time of each MT intervention post-discharge were not specified in five studies [59,61,64,65,66]; nonetheless, four of these followed the LongSTEP protocol in which MT post-hospitalisation commenced shortly after discharge. In other studies, the age of preterm-born children spanned from two months corrected age (CA) to 24 months [44,45,46,60,62,63,67]. Full term infants also participated in the two earliest studies [44,45], with the most recent research studies only comprising preterm infants [46,59,60,61,62,63,64,65,66,67], although one study did include typically developing infants in their group MT sessions [46].

Four studies did not provide any information on the ages of the adult participants [44,45,46,65]. In LongSTEP articles with greater participant numbers, parental ages were presented as mean years [64,66], while other studies specified exact ages [61,63,67] or an age range [59,60,62]. Mothers were involved in each research project [44,45,46,59,60,61,62,63,64,65,66,67], whereas fathers did not participate in three studies [44,46,61,63]. In one study, participants included fathers, grandparents, and children’s nannies; however, it was unclear if they were caregivers of preterm or full term infants and toddlers [45], as no specific participant details were given. In contrast, fathers participated in all LongSTEP research studies [59,60,62,64,65,66], albeit to a lesser degree than mothers. The treatment delivery settings varied from outpatient hospital spaces and MT clinics to family homes [44,45,46,59,60,61,62,63,64,65,66,67].

Of the 12 research study designs, five were quantitative [44,45,46,64,66], five were qualitative [61,62,63,65,67], and two feasibility studies combined quantitative and qualitative methods [59,60]. Three qualitative [61,62,63] and three quantitative [44,45,46] studies focused on MT with preterm infants and their families during the post-NICU discharge period; however, of these, two quantitative studies also included at term-born children, with no definitive results related to preterm infants [44,45]. The remaining six studies all comprised MT with families in the NICU followed by MT post-hospital discharge [59,60,64,65,66,67].

Interventions and therapeutic techniques varied according to each study’s aims, methodology, and study design. For example, group-based studies using the Bright Start curriculum [44,45,46] included a range of therapeutic activities, such as playing instruments, singing, exploring picture books, and movement to music. Conversely, individual MT sessions in the LongSTEP study used interventions in which parental voice was the focal point for achieving connection between preterm infants and their caregivers [59,60,62,64,65,66,67]. Similarly, therapeutic vocal interventions were employed in the CoPE with music [62] and Reflective Lullaby Writing [64] studies as a means for mothers to connect with their babies as well as with their own physical and symbolic voice during individual MT.

In some studies, MT occurred with greater regularity in a shorter time frame, such as twice-weekly or weekly group-based MT [44,45], or individual weekly MT for eight weeks [61], whereas other studies delivered fortnightly or monthly sessions over a 6-month period [59,60,62,63,64,65,66,67]. The frequency of attendance varied, with one group-based quantitative study permitting participation in a single MT session [46], whereas engagement in a minimum three or four sessions post-discharge was mandatory for other quantitative research’s group music classes [44,45]. For individual-based quantitative LongSTEP studies, it was mandatory to attend six of seven sessions. Qualitative [61,62,63,65,67] and feasibility studies [59,60] differed in their frequency, from two to eight sessions. The time length of group-based MT sessions varied from 25 to 45 min [44,45,46], whereas individual MT ranged from 45 to 90 min [59,60,61,62,63,64,65,66,67].

The quantitative studies in this review primarily employed questionnaire formats to gather data, either using validated tools (psychometric [45,46,66], demographic [45]), and/or measures created specifically for MT research purposes [40,45,64]. Audio and/or video data from MT sessions were collected in three quantitative investigations [40,45,64]. Quantitative data were analysed using either inferential [40,41,42,43,44,45,46,64,66] or descriptive [45,64,66] statistics.

In four qualitative studies, researchers collected data via audio and/or video recordings of MT sessions [61,62,65,67]. Of these, the three LongSTEP studies also used semi-structured interviews for further data collection, with all data analysed via Interpretative Phenomenological Analysis (IPA) [65,66,67]. The CoPE with music researchers transcribed each session as well as notating any music made, for example, improvised songs and infant vocalisations, with data explored via content analysis [61]. One study collected qualitative data via audio/video-taped semi-structured interviews [63], with analysis via McFerran and Grocke’s Microanalysis in Music Therapy method [80]. Of the nine studies focusing on the impact of MT on parents of preterm infants and/or their perceptions of MT, the majority of data were collected from mothers [45,59,60,61,62,63,65,66,67].

Two LongSTEP feasibility projects were the only studies using both quantitative and qualitative measures for data collection [63,64]. However, due to insufficient participant numbers, one study did not analyse data using quantitative or qualitative methods, instead reporting quantitative trends and qualitative citations of participant comments [59]. The other study used descriptive statistics and inductive thematic analysis [60].

Quantitative results ranged from trends and significant findings in all group-based MT studies [44,45,46] to no clinically important effects in the main LongSTEP trial [66]. All qualitative studies detailed parental experiences of, and reflections on, MT post-discharge, with the majority of parents evaluating it positively [61,62,63,65,67] and a minority acknowledging challenges [62,65]. The two feasibility studies produced both quantitative and qualitative findings that confirmed the feasibility of implementing the LongSTEP protocol [59,60], whereas the quantitative study investigating LongSTEP’s treatment fidelity suggested it was applicable to multisite settings worldwide [64].

### 3.1. Themes

#### 3.1.1. Theme 1. Creating Supportive Environments

The majority of articles discussed how MT supported families to: (a) adapt and adjust to life after hospital discharge, (b) express emotions and feelings related to having a child/children born preterm, or (c) calm or engage themselves and/or their preterm babies [59,60,61,62,63,65,66,67]. A MAXQDA word frequency count revealed the word stem ‘support*’ was absent in early articles [44,45,46], minimally used in three LongSTEP studies [59,64,66], and frequently found in all remaining publications [60,61,62,63,65,67]. It was associated with encouraging musical engagement [60,63,65,66,67], helping parents develop their parental roles [60,61,62,63], promoting peer support for LongSTEP music therapists [64], and parent–infant bonding [60,62,63,65,66,67]. Eight studies referred to the supportive environments or spaces MT provided post-discharge, whether physical [45,61,63,65] or metaphorical [60,61,62,63,64,65,67].

*Sub-theme 1: A Physical or Metaphorical Space*

Physical environments varied according to study aims, with Walworth’s [45] research requiring a large room for developmental group activities, whereas the CoPE (Communicative Parental Efficacy) with music method created a calm, nest-like setting at a MT university clinic for a mother and her preterm son [61]. Both LongSTEP feasibility studies conducted MT in various spaces based on parental preference (family home, MT centre, and/or hospital) [59,60]. Some articles mentioned the benefits of MT commencing in the NICU and continuing after hospital discharge. Two LongSTEP articles confirmed that post-discharge MT helped families adapt from the hospital to home environment more easily via a continued therapeutic connection with their NICU music therapist, illustrating MT’s potential benefits during critical transition periods [59,65]. Other research focused exclusively on post-NICU discharge MT programs. Both Calderon-Noy and Gilboa [61] and Howden et al. [63] found individual MT sessions assisted mothers in adjusting to life with their preterm infants at home, with the therapeutic relationship between the music therapist and themselves providing a metaphorical supportive space in which this occurred.

One study portrayed MT as both a physical and figurative sanctuary, offering parents respite from everyday life after hospital discharge, a space to acquire resources, and a place to contemplate their experiences of preterm birth and life thereafter [65]. Some studies further highlighted nurturing and therapeutic musical spaces, with one mother perceiving MT as akin to being “on an island surrounded by a stormy sea” [67] (p. 1). Another music therapist established a trusting therapeutic environment with two mothers by supporting parent–infant musical interactions prior to lullaby creation [63]. This inspired one of them to set up a physical space where she and her son could engage in MT and other creative activities together, while enabling her to integrate a growing musicality into daily life with her infant son [63]. Equally, participation in MT sessions resulted in other parents learning how to incorporate music into everyday life with their preterm infants, such as during nappy (diaper) changes, bath time, emotional regulation when unsettled, and play time [60,61,62,64,65,66,67]. For other parents who had engaged in MT both in the NICU and post -discharge, MT became a creative, connecting bridge between the hospital and life at home after discharge [65].

*Sub-theme 2: A Space for Expression, Insight and Reflection*

Certain studies referred to MT as a space in which parents were able to express feelings and emotions related to their recent experiences of preterm birth, hospitalisation, discharge to home, or life in general. One mother gained a deeper understanding of herself via the therapeutic process of lullaby songwriting, acknowledging personal trauma and rediscovering past coping strategies in one-to-one MT sessions [63]. Correspondingly, another participant in the same research project reconnected with an interest in musical self-expression, particularly singing, which increased her confidence in her parental identity [63]. Parents from the Israeli arm of the LongSTEP study recognised MT as a space to express creativity, process their experiences of preterm birth, and speak freely [62,65,67]. Some parents noted the necessity for supportive dialogues with the music therapist before engaging in musical interactions with their infants during MT [62]. Another mother gained personal insights during discussions with the music therapist, which resulted in increased self-expression at home and greater parental confidence when interacting with, and caring for, her baby [61]. All parents in one Israeli LongSTEP study spoke about experiencing a sense of freedom when singing at home compared to the NICU [65]. Online spaces were also mentioned, with mothers in one study identifying the benefits of completing a song via Zoom during COVID-19 lockdowns [63]. A further study commented on the supportive online space LongSTEP supervisors offered to 11 music therapists delivering MT sessions worldwide, allowing them to discuss successes or challenges, exchange ideas regarding protocol implementation, and engage in self-reflection [64].

*Sub-theme 3: A Space of Calmness or Engagement*

Some review articles alluded to MT’s calming effects post-hospital discharge both during and after MT. In the Polish LongSTEP feasibility study, a mother reported increased tranquility for her twin babies and herself after their first home-based MT session, with the infants also becoming calmer after subsequent MT sessions [59]. These findings align with other LongSTEP-based studies. For instance, one mother observed her ability to soothe both her daughter, and herself, during difficult times after participating in post-discharge MT [65,67]. Other parents noted their babies’ transitioning from distressed to calm states with parental singing [65] or humming [60], while an entire family engaged in group singing to comfort their youngest preterm child during a car trip [62]. Equally, a mother was able to settle her baby at home, and during a vaccination appointment, using techniques from the CoPE with music method [61].

MT also facilitated participant engagement, with structured, music therapist-led group MT sessions encouraging social behaviours between infants and toddlers [44,46], and infants and their caregivers [44,45,46]. Individual family-centred MT sessions in some LongSTEP studies reported similar findings; two mothers noted high levels of participation from their baby twin boys during MT at home [59,65]. Other parents acknowledged their infants’ vocal and gestural communication during MT as positive feedback for parental singing, leading to increased musical involvement with their infants both in MT sessions [60] and in daily life [60,62,65]. An Australian study found individual MT sessions provided a space for mothers to musically interact with their babies and explore their own musical creativity [63]. In a quantitative study assessing LongSTEP’s treatment fidelity, external raters and music therapists rated parents’ musical engagement (as observed on video) much lower than the parents did themselves [64]. Conversely, a qualitative Israeli study reported parents leading musical interactions with their infants during MT post-discharge, as per the LongSTEP protocol [62].

#### 3.1.2. Theme 2. Developing Skills, Tools and Resources

All articles discussed the role of MT post-hospital discharge in helping preterm infants and their families develop skills, tools, and resources. A MAXQDA word frequency analysis found the term ‘skill’ most frequently in two journal articles: Standley et al. [44] and Epstein et al. [62]. Each study had divergent objectives, with Standley et al.’s quantitative behavioural approach aimed at infants and toddlers achieving a measurable skillset, whereas Epstein et al. focused on parents expanding and enhancing their musical and communication skills. The term ‘tool’ was present in five qualitative studies, referring either to the musical and communication techniques parents learned during post-discharge MT [61,62,63,65] or a music therapist’s understanding of the construct ‘agency’ [67]. Most studies mentioned the term ‘resource’ at least once, ranging from Howden et al. referencing a mother’s “personal resources” [63] (p. 15) to all LongSTEP articles frequently discussing ‘resources’, ‘resourcefulness’, or ‘resource-oriented’ perspectives in the context of MT sessions. These primarily referred to parental resources, including emotional, inner, musical, nurturing, parenting, or vocal resources [59,60,62,64,65,66,67]. The term ‘resource’ was absent in the CoPE with music study [61] and studies prioritising infants’ acquisition of developmental skills via group MT [44,45,46].

*Sub-theme 1: Infants*

The earliest studies noted that both preterm and typically developing infants and toddlers scored higher in diverse developmental skills compared to control groups after engaging in structured group MT programs based on the Bright Start curriculum [81]. A pilot study demonstrated that toddlers (12 to 24 months) attending four to seven MT sessions had higher cognitive, but not social/motor, skill scores when compared to a control group. Increased session attendance correlated positively with higher scores [44]. Scores also indicated a trend towards higher levels of developmental delay in preterm infants (*n* = 3). In contrast, a later study using an adapted version of Bright Start determined improvements in 12-month scores for motor, cognitive, and communication skills [46] among younger preterm infants at high risk of neurodevelopmental delay (6–12 months), surprisingly including those who had only attended one MT session. Contrary to Standley et al.’s [44] results, Walworth [45] found that preterm infants and toddlers demonstrated higher-level social skills than full term controls during toy play with their caregivers.

The main LongSTEP trial reported no clinically significant effects related to the acquisition of infant development skills in preterm infants who had participated in individual MT post-NICU discharge [66]. Despite this, qualitative data from both the LongSTEP trial and other studies included parents’ observations of their infants developing skills in areas such as cognition [59,61,62] communication [60,62,65], motor skills [60,61], and socialisation [60]. One LongSTEP-based study also proposed that infants may have accessed resources via musical interaction and co-regulation with their parents during post-discharge MT [67].

*Sub-theme 2: Parents*

Ten publications documented parents developing skills and using tools and resources during, or as a result of, MT sessions post-hospital discharge [45,59,60,61,62,63,64,65,66,67]. One quantitative study explored the effects of the Bright Start program on parental responsiveness, reporting a trend (albeit not statistically significant) toward greater responsiveness in the experimental group parents to their children’s social cues during toy play compared to a control group [45]. Similar to fathers in the Polish LongSTEP feasibility study [59], parents from the Norwegian LongSTEP feasibility study expressed appreciation for post-discharge MT, transferring skills and resources acquired during hospital-based MT to home and, for example, singing/adapting songs for feeding or transition to sleep [60]. Norwegian parents also reported a higher aptitude in reading infant cues due to post-discharge MT participation [60]. Four Israeli mothers stated that MT offered helpful musical coping tools for interpreting infant behaviour, communicating, and/or handling challenges with their babies post-discharge [61,62,65,67].

Music therapists employing the resource-oriented LongSTEP MT approach also supported parents to use their own musical and nurturing resources in a developmentally appropriate way when interacting with their preterm infants [59]. Four LongSTEP studies highlighted the enhancement of parent–infant communication skills in daily life with their infants, with certain parents recognising music—and others their own voices—as key resources [62,64,65,67]. Three LongSTEP studies noted resource-oriented MT’s impact on participants’ sense of agency, suggesting an evolving musical agency could enhance personal and parental agency [62,65,67]. Not all parents experienced a transformative effect, with one mother finding that singing to her twins was a normal activity unrelated to MT [62], and the main LongSTEP study’s quantitative results showing no benefits for parents from the potential skills and resources attained during MT in the NICU and/or post-discharge [66].

In other studies, the acquisition of skills, tools, and resources augmented parental confidence and competence, thereby offering additional opportunities for change and development in parents’ interactions with their preterm babies after hospital discharge. The Reflective Lullaby Writing intervention encouraged two mothers to contemplate their personal resources, as well as build a musical and/or parental identity. This assisted them to transform the trauma arising from preterm birth and hospitalisation into positive change, resulting in an increased ability to build relationships with their infants [63]. Similarly, the CoPE with music method increased one mother’s ability to care for her infant, with the music therapist modelling vocal soothing and/or interaction techniques before supporting the mother in practising them during MT with her infant. This enabled her to apply these communicative parental efficacy skills in daily life by voicing opinions in the face of family criticism, understanding her son’s perspective, and connecting with him through vocalising and singing [61].

*Sub-theme 3: Music Therapists*

The skills required of music therapists working in the post-NICU discharge environment have evolved from those of a therapeutic expert to those of a therapeutic support person. Earlier studies focused on music therapists conducting highly structured, group-based MT sessions [44] and instructing, training, or coaching parents on how to use developmentally appropriate musical activities during MT and at home [44,45,46]. In contrast, recent studies have emphasised the music therapist’s role in supporting parents to become the experts during individual MT sessions. This involved the music therapist forming a therapeutic relationship with parents [63,65,67]; offering psychotherapeutic assistance by discussing parental concerns [60,61,62,63,67]; modelling, guiding, and/or collaborating in parent–infant musical interactions [59,60,61,62,63,64,65,66,67]; encouraging infant, parental, and musical agency [62,65,67]; offering musical and/or vocal accompaniment [62,63,65,66,67]; and promoting bonding [59,60,62,65,66,67] by following the natural flow of interactions between parent–infant dyads/triads [60,61]. Interestingly, one LongSTEP-based case study proposed that individual parent preferences should guide who leads the musical engagement in post-discharge MT sessions, even if this results in the music therapist providing more directive support than the program protocol advises [67]. Additionally, this study accentuated musical agency as a developing, collaborative process, or creative exchange, between infants, parents, and the music therapist, rather than something that originated solely from the therapist [67].

#### 3.1.3. Theme 3. Acknowledging Challenges

Most research studies have mentioned challenges related to implementing MT programs with preterm infants and their families after hospital discharge. A MAXQDA word frequency count discovered the word stem ‘challeng*’ in five studies [59,60,61,62,64,67] and most frequently in the LongSTEP feasibility studies [59,60]. Researchers also searched for ‘challenge’ synonyms, finding words deriving from the core terms ‘difficult*’ in six studies [59,60,61,63,65,67] and ‘barrier’ in one study [62].

*Sub-theme 1: Infant Challenges*

Infant challenges were seldom mentioned, although a few specific difficulties were highlighted. In the earliest study, toddlers were reluctant to vocalise during their first group MT session. This was attributed to their unfamiliarity with the group setting and resolved as sessions progressed [44]. Another challenge was parents struggling to connect with their infants and/or understand their communication cues. However, post-discharge MT interventions encouraged babies to respond to, and initiate interactions with, their parents, resulting in increased parent–infant dialogues [61,62,67]. One baby also struggled with the unaccustomed sound of a guitar during post-discharge LongSTEP MT sessions, resulting in his parents learning to adjust their behaviour to his individual reactions [60].

*Sub-Theme 2: Parent Challenges*

The most commonly reported parental challenges were divided into three categories: emotional difficulties, reluctance to sing, and paternal absences. Some parents experiencing emotional challenges needed a supportive discussion with the music therapist before being able to musically engage with their baby [61,62]. One mother addressed various emotional issues during reflective lullaby writing, acknowledging that the therapeutic environment provided by the music therapist was crucial to her wellbeing [63]. Despite valuing MT post-discharge, another mother, who had already participated in hospital-based MT, associated it with painful and emotional memories of the NICU [65]. One family declined to continue MT after NICU discharge as they were burdened by the demands of caring for their preterm baby and her older siblings [60].

Some parents expressed embarrassment about singing in front of the music therapist during post-discharge MT. For instance, none of the parents in one LongSTEP study had participated in NICU MT, resulting in an initial reluctance to sing; nevertheless, this decreased to varying degrees as the sessions progressed. The study also offered recommendations for sensitively addressing such issues in the future by adapting post-discharge MT to each family’s individual needs, ensuring a more individualised and effective approach [62]. Another LongSTEP article reported similar issues and outcomes for parents during NICU-based MT, with some nevertheless experiencing an increased sense of freedom when singing to their infants at home after hospital discharge [65].

Fathers were absent in 5 out of 12 studies [44,46,61,63,67]. In the remaining studies, participation varied: one did not specify the exact number of paternal participants [45], two included equal numbers of fathers and mothers [59,60], and four comprised fewer fathers than mothers [62,64,65,66]. A surprising outcome was that one father who had not participated in the CoPE with music program nonetheless benefitted from his wife’s participation, with her new ability to express emotions at home leading to a similar change in his behaviour [61].

*Sub-theme 3: Research Challenges*

Research challenges included accessing sufficient preterm participant numbers for quantitative data collection and analysis [44,45,59,60], parents self-selecting their participation in the experimental or control groups [45], the limitations of a quantitative software analysis tool [45], and a retrospective study design ruling out causal inferences [46]. Other challenges comprised the lack of a control group [59,60], having to translate research instruments into another language [59], dual roles as clinician–researcher [60,61,62,63,64,65,67], low-quality audio/video recordings for analysis [64], and the potential low sensitivity of a psychometric instrument [66]. The COVID-19 pandemic also impacted session formats, session locations [63,66], and the researchers’ capacity to interpret and reflect upon data [63,64]. Additionally, logistical challenges involving time limitations, scheduling post-discharge MT sessions, and limited MT resources were noted [59,60]. The LongSTEP study faced the specific challenge of creating a protocol that ensured both standardised implementation across multiple research sites and the ability to accommodate any cultural and family-specific adaptations [64].

#### 3.1.4. Theme 4. Building Relationships

The majority of studies emphasised aspects of building relationships within a MT framework post-hospital discharge. The term ‘relationship’ was found in all studies besides Standley et al. [44], with a MAXQDA word frequency count revealing the highest incidence in articles by Epstein et al. [62], Epstein et al. [65], and Epstein [67]. The word stem ‘communicat*’ occurred most consistently in research studies by Calderon-Noy and Gilboa [61] and Epstein et al. [62], primarily referring to parent–infant communication. Words deriving from the core term ‘connect’ were absent from all quantitative studies [44,45,46,64,66] and the Polish LongSTEP feasibility study [59], but frequent in qualitative studies [61,62,63,65,67] and the Norwegian LongSTEP feasibility study [60].

*Sub-theme 1: Infants and Parents*

The most frequently encountered sub-theme involved building relationships between infants and parents and was divided into three categories: infants, parents, and parent–infant bonding. The majority of studies underscored the infants’ ability to initiate and respond to communication with their caregivers [45,46,59,60,61,62,63,65,67]. For instance, Bright Start participants (preterm/full term) scored higher in socialisation and communication compared to full term controls [45,46]. The qualitative and feasibility studies mentioned MT’s impact as well, with mothers in one study noting that their infants’ ability to babble, make eye contact, and interact developed after participating in MT post-discharge [60]. Another mother from the same study observed her son demonstrating a greater interest in using his voice to communicate compared to infants of a similar developmental age, though she expressed that it may have been coincidental [60].

Parents in some studies were inspired to communicate with their babies after observing their reactions to the music therapist modelling vocal dialogues [61,62]. A father from one LongSTEP study reported participation in post-discharge MT had enabled him to clearly recognise his baby’s musical communication responses [62]. Each parent in this study also acknowledged their babies’ reactions as the key motivator for engaging in musical dialogues. Similarly, parents from another LongSTEP study observed their infants’ transition from responding to music to becoming musical collaborators, encouraging parents to create music with them both during MT and in daily interactions. After the MT program concluded, these participants continued to integrate music into home life at their infants’ request [65]. This corresponds to findings from Howden et al.’s study, in which a mother reflected that her son’s responses during parent–infant musical interactions in MT had inspired her to continue singing and playing music according to her son’s behaviour and cues in daily life [63].

One quantitative study reported no significant differences in parent–child interactions after attending group MT, although there was a trend towards parents in the experimental group showing more positive responses to their children’s cues during toy play [45] than the control group. However, as there were no specific data on the parents of preterm infants, no correlation could be made between parent and infant responsiveness with this cohort [45]. The Polish LongSTEP study noted how fathers communicated and interacted with their preterm infants in post-discharge MT via songs learned while observing, but not participating in, MT in the NICU [59]. Other studies reported the continuation of parental singing and music making at home, both during a post-discharge MT program and afterwards [60,61,62,63,65,67]. Parents in the Norwegian LongSTEP feasibility study reflected on the significance of their singing voices in promoting communication with their infants, particularly post-discharge [60]. Equally, parents from three Israeli LongSTEP studies acknowledged that the vocal interaction and singing skills acquired during MT had increased their ability to communicate creatively with their infants post-discharge via co-created vocal/musical dialogues [62,65,67].

Several studies explored the impact of MT on parent–infant bonding. Quantitative data from the LongSTEP trial found that parent-led MT had no clinically significant effect on mother–infant bonding as measured by the Postpartum Bonding Questionnaire (PBQ) [66]. These findings contrast with data from other qualitative LongSTEP studies in which parents reported that MT helped them to build relationships and bond with their preterm-born children post-discharge. One mother specifically noted a stronger connection with her preterm baby in later sessions compared to the introductory session [62]. Another mother confirmed that active engagement in MT had strengthened the bond with her twin boys [59]. Likewise, participants in Epstein et al.’s LongSTEP study [65] frequently verbalised how MT offered opportunities to bond with their preterm babies [65], with Epstein’s case study not only providing further confirmation of this but also introducing the possibility of a link between musical agency and bonding [67]. Standley et al. [44] and Hamm et al. [46] outlined musical activities to promote bonding; however, they did not provide any quantitative results on their effectiveness for caregiver–infant dyads. In contrast, Howden et al.’s research [63] offered an outline of the two-phase therapeutic intervention being piloted, with comprehensive qualitative data for analysis and insights reinforcing Gaden et al.’s [82] research recommendations that it may be more beneficial for music therapists to support parent–infant bonding once a family has been discharged from hospital.

*Sub-theme 2: Infants and Others*

Studies in this integrative review occasionally referred to MT facilitating communication and connections between infants and other family members. Examples included one preterm infant from a LongSTEP feasibility study responding to his older siblings’ musical interactions with loud singing [60]. In another LongSTEP study, older siblings sang with both parents to soothe their distressed, younger, preterm brother [62]. Indirect benefits from one mother’s participation in the CoPE with music study were also noted, with one elderly woman motivated to interact musically with her great-grandson after observing his mother communicating with him via singing and improvised vocalisations [61].

Walworth [45] mentioned the attendance of grandparents and children’s nannies during MT; however, as data were not separated into specific caregiver cohorts, it was not possible to draw conclusions regarding the effects of MT on their responsiveness. Similarly, two other studies briefly noted the participation of older sisters during MT sessions, but further information was unavailable [59,60]. Several studies also alluded to infants engaging in therapeutic relationships with music therapists, primarily through vocal interactions, in which the music therapist modelled musical conversation [61]. Likewise, the music therapist’s role in the LongSTEP approach comprised supporting the parent–infant relationship via an initial modelling of therapeutic musical interactions [60,61,62,65,67].

*Sub-theme 3: Parents and Others*

Qualitative post-discharge MT research has emphasised the development of therapeutic rapport between parents and the music therapist. In the Reflective Lullaby Writing study, an Australian music therapist provided a space for two women to verbalise and contemplate the significance of being mothers of preterm children [63], enabling them to write an individual lullaby about these experiences. Another mother and MT clinician researcher in the LongSTEP study established a partnership through singing together as well as exchanging thoughts and observations related to the preterm baby’s behaviour and needs [67]. The music therapist from the CoPE with music study employed similar therapeutic techniques, resulting in a significant improvement in the mother’s confidence when communicating with, and caring for, her preterm baby at home.

Several parents acknowledged that the music therapist’s musical and vocal contributions were crucial to feeling supported when singing to their infant/s during MT, whereas other participants appreciated the music therapist leaving space for them to sing independently, as this contributed to their own growth in confidence [62]. Some parents even experienced a sense of connection, or relationship, with other entities, such as their own voice [62,63], a musical instrument [62], or a lost part of themselves [63], through engaging in MT post-NICU discharge.

## 4. Discussion

This review suggests that MT supported preterm infants and their families post-hospital discharge, albeit with further research required to address discrepancies between quantitative and qualitative findings. We identified research studies, established the similarities and differences in clinical approaches, synthesised the themes arising, and discovered gaps in knowledge. In summary, the professional literature on this topic is still limited, with seven studies referencing the LongSTEP trial [59,60,62,64,65,66,67], three based on the Bright Start curriculum [44,45,46], and two innovative programs in case study [61] and pilot formats [63].

Recent studies have pivoted from evaluating MT’s effects on preterm infant development to determining its impact on parent–infant relationships, possibly influenced by evidence linking parents’ mental and physical health post-discharge to their preterm infants’ development [8,11,83]. Research has also shifted from including term-born infants/toddlers [44,45,46] to focusing exclusively on preterm participants [59,60,61,62,63,64,65,66,67], presumably due to increased MT services for premature babies and their families over the past 30 years [84] and the corresponding higher availability of preterm infants for research studies.

To our knowledge, this is the first literature review examining MT research with preterm infants and their families post-discharge. Due to the limited number of published studies, we could not compare or contrast our findings with additional literature from the post-NICU discharge MT field in the following discussion. Instead, we incorporated research from NICU MT, early childhood MT, and other health disciplines to do so. The key areas of our identified themes will be discussed below. 

### 4.1. Supporting Families in Creative Spaces

Most reviewed articles reported that MT assisted parents’ adaptation from the hospital to home [59,61,62,63,65,67]. Likewise, an interdisciplinary care team (including MT) provided parents with continuity of care, growth in self-confidence, and emotional support in a study investigating the “Transition to Home” (TtH) model, although MT was only offered in the NICU, with home visits conducted by an advanced practice nurse (APN). Several parents who had participated in NICU MT continued to use singing and music as a calming tool at home for themselves and their babies [85], leading us to query if this degree of MT support might suffice for some families, while others, such as the parents of preterm infants at high risk of developmental delay, may benefit from MT sessions after discharge.

In this integrative review, some publications also referred to MT creating supportive metaphoric places [65,67], physical environments [61,63], and musical spaces [63] for preterm infants and their families post-discharge. Other studies on NICU and paediatric intensive care unit (PICU) post-discharge care have focused more on the role of individual post-discharge interventions or health care clinicians offering psychosocial assistance in follow-up clinics or at home [86,87]. For some parents in this IR, post-discharge MT even served as a nurturing, metaphoric bridge between the neonatal unit and home [62,63,65]. The same construct has featured in other research, where various healthcare clinicians, including a music therapist [34], APN [85], occupational therapist [30], and paediatric nurse specialist [88], assisted families in the transition from the NICU to home.

Parents in the IR studies developed a deeper understanding of the emotional impact of caring for a preterm infant and discovered innovative, and often musical, coping strategies [60,61,62,63,65]. This may be due to a sense of competency [59,60,61,62,63,65,67] and freedom [61,65] experienced by some parents in MT sessions, which aligns with an educational study confirming that children and young people developed insights into, and competencies in, academic and social areas of their lives through music education activities that offered creative choices and learning opportunities [89]. We suggest this capability approach may be associated with communicative music efficacy [61], parental confidence [63], resource-oriented MT [59,60,62,64,65,66,67], and personal and/or parental agency growing in tandem with musical agency [65,67].

Our review found that music had calming effects, with mothers learning vocal techniques to calm both their babies and themselves during post-discharge MT [59,61,65]. This is substantiated by research demonstrating that mother-sung lullabies during infant immunisation significantly decreased infant pain responses and maternal anxiety [90,91,92]. A comparable outcome was observed with parental infant-directed singing during painful procedures in the NICU [93,94]. This review also highlighted MT’s engaging impact, with parents noting enriched parent–infant and infant–parent interactions in daily life as a result of MT participation [59,60,62,65,67], with similar results found in research into early childhood MT programs [95,96].

### 4.2. Strengthening Competence and Capability

The earliest quantitative studies in this review revealed positive outcomes for preterm infants participating in group MT [44,45,46], aligning with findings from *Sing&Grow*, a group-based early childhood MT program, which identified significant improvements in children’s behavioural, cognitive, motor, and social skills [97]. Surprisingly, recent quantitative findings from the LongSTEP trial demonstrated that individual MT had no significant effects on infants or parents [66], while qualitative data indicated the opposite [59,60,62,63,65,67]. This discrepancy suggests that integrating both quantitative and qualitative results, as proposed by Slonim-Nevo and Nevo [98], might provide a more comprehensive understanding of MT’s impact. Ghetti et al. [66] also proposed that additional LongSTEP MT sessions may have contributed to more conclusive infant development results. Nevertheless, qualitative data from the CoPE with music case study [61], Howden et al.’s research [63], and five LongSTEP studies [59,60,62,65,67] indicated that parents acquired competencies (musical or otherwise) during post-discharge MT, with which they managed their infant’s care and/or connected with them in daily life. This is consistent with research into other supportive MT programs, like Serenade, where parents of both typically developing and autistic preschoolers reported improved parenting skills via musical strategies learned in MT [99]. Similar benefits were observed in early childhood studies, where one father described the *Kindermusik* program as a play/bonding tool [100], while other parents gained confidence by using remote monitoring technology to manage their children’s diabetes [101].

Qualitative data related to post-discharge MT [59,60,61,62,63,65,67] also highlighted the interdependent nature of parents developing competence and capability and corresponding improvements in infant development. Likewise, a review of home-based post-discharge programs for parents of preterm and high-risk infants concluded that early parent–child interventions supporting parental responsivity also resulted in infant adaptation, resilience, and competence [102]. Comparably, participation in a parent-implemented EI program for autistic toddlers resulted in significantly improved parent interaction skills compared to a control group and infant developmental improvements [103].

Some review articles emphasised a resource-oriented MT approach, with parents developing musical and communication skills that enhanced their sense of parental and musical agency [59,60,62,64,65,66,67]. This parallels other contexts, where resource-oriented strategies have promoted the agency of parents of children with life-limiting illnesses [104], addressed linguistic diversity in primary school classrooms [105], and undertaken collaborative, empathetic MT assessments in child and youth mental health settings [106]. In this IR, parents developed parental confidence [63], identity [62,63] self-efficacy [61], and musical agency through connecting to their own voices via singing [59,60,64,65]. This corresponds to significant improvements in parental self-efficacy upon attending the previously mentioned *Sing&Grow* [96] MT program. However, not all parents in the IR studies experienced increased parental and musical agency post-discharge [62]. This may have been proportionate to parental ease, as the only parent expressing an ongoing reluctance to sing during MT also perceived music’s contribution to her parenting as insignificant. This accords with another review suggesting that active engagement, or ‘embodied doing’, in EI programs may enhance parental competence, as opposed to parents who remained passive or uncomfortable [107].

Both clinical practice and research into MT post-NICU discharge have evolved, with this review underscoring the evolution of a music therapist’s role from expert clinician leading group classes [44,45,46] to musical collaborator supporting parents in individual sessions [59,60,61,62,63,64,65,66,67]. Globally, neonatal MT appears to have undergone a similar transition, possibly influenced by family-centred care principles advocating for greater parental involvement in their preterm infants’ care [108,109]. In the past three decades, research that once focused on the effect of recorded music [110,111,112,113,114] or direct music therapist–infant interactions [115,116] now emphasises supporting the parent–preterm infant dyad/triad [117,118,119,120,121,122,123,124,125,126].

### 4.3. Recognising Barriers

This review identified various barriers to post-discharge MT, such as some parents needing supportive discussions and/or counselling before being able to participate [61,62,63,65]. Likewise, a meta-analysis of EI programs for preterm infants confirmed the importance of health care professionals providing emotional assistance for parents [127]. Parental peer support initiatives have also shown promise in previous neonatal research; however, their effects on mental health outcomes post-NICU discharge remain inconsistent [128,129,130]. Our IR findings indicate that MT generally helped parents adjust to life after NICU discharge [59,60,61,62,63,64,65], although one family felt overwhelmed by caring responsibilities and opted out [60]. This suggests that some families may require customised psychosocial support and/or more time to adapt to changes in their home life before being offered MT. Other families may find other forms of emotional support more appropriate than MT. External factors, such as local customs [131], sociocultural biases [132], or legal fears among undocumented parents [133], also influence the feasibility of post-NICU therapies. For instance, cultural norms in some communities assign childcare solely to mothers, impacting fathers’ participation [131]. We suggest music therapists working with families after hospital discharge familiarise themselves with the abovementioned, and other, barriers to either work around them or understand why their offer of therapy may be declined.

According to this review, some parents were hesitant about singing and/or playing music during individual MT after hospital discharge [62,65], with music therapists urged to consider this prior to commencing sessions with each family. Observing a music therapist model vocal interactions was effective in decreasing parental embarrassment, as per the CoPE with music program and LongSTEP trial [60,61,62]. Other MT research with vulnerable first-time parents also confirmed a reluctance to sing at the beginning of antenatal group sessions or individual home visits, which nonetheless diminished with the building of trust between young parents and the music therapist [134]. Alternatively, shared reading [135] or guided play [136] might better suit some families.

Although some studies included fathers [59,60,62,65], post-discharge MT research typically focused on the mother–infant dyad, with a resultant knowledge gap regarding paternal involvement. In the broader literature, paternal perspectives are increasingly represented post-discharge, with one study finding that journaling or blogging provided an expressive outlet for fathers after NICU discharge [137], and another showing that the NICU2HOME app helped fathers gain self-efficacy in the first 30 days after discharge [138]. Telehealth MT may also be an option for fathers restricted by work or distance, or for families who live too far away from health care services, as evidenced by its acceptance in regional Australia for physiotherapy with preterm infants [139]. Telehealth has also proven effective in reducing maternal stress/anxiety and increasing mother–infant attachment in tele-homecare settings with the families of preterm infants [140].

Research challenges included the integrating of full-term and preterm infant data in early studies, with insufficient preterm participant data available for a separate analysis [44,45]. The LongSTEP trial, however, managed to gather sufficient data by employing a multisite approach and focusing on treatment fidelity to accommodate cultural and family-specific needs [64,66]. Likewise, other multisite trials facing similar issues have engaged in comparable treatment fidelity assessment practices [141].

### 4.4. Establishing Rapport

This review found that participating in MT post-discharge enhanced preterm infants’ ability to respond to and communicate with their parents [45,46,59,60,61,62,63,65,67]. A significant factor was parents’ continued use of MT vocal techniques in daily life, motivated by their infants’ positive reactions [61,62,63]. Similarly, a scoping review of preterm language development after NICU discharge indicated that caregivers who received guidance on parent responsiveness and child development showed increased sensitivity to their children’s responses, leading to improved language and cognitive development in preterm infants at six and 12 months [142]. The effects of parental singing on parent–infant bonding demonstrated mixed results. Quantitative data indicated that MT had no effect on mother–infant bonding during or after NICU hospitalisation [66], while qualitative data suggested otherwise [59,60,62,65]. This discrepancy correlates with findings from other studies; for instance, live maternal lullaby singing increased postnatal bonding between mothers and babies born at term [143], whereas recording songs for preterm or full-term infants did not significantly enhance mother–infant bonding [144]. As Ghetti et al. [66] recommended, further investigation is warranted into the longer-term effects of MT on parent–infant bonding and parental wellbeing. Qualitative evidence highlighted family communication and interactions beyond the mother–infant dyad post-discharge, including fathers and siblings’ contributions during and after MT sessions [59,60,62]. While in no way diminishing the integral role mothers play in the care of preterm infants [145,146,147], incorporating other family members into MT research post-hospital discharge is crucial to promoting inclusive research practices, comparable to those in recent NICU-based MT studies [20,37,148,149,150,151,152,153].

A consistent theme in this IR involved establishing and maintaining a therapeutic rapport between music therapists and parents, particularly in qualitative studies based on the LongSTEP method [62,65,67], the CoPE with music program [61], and the Reflective Lullaby Writing pilot study [63]. These studies emphasised a supportive, collaborative relationship for the benefit of either a parent, parent–infant dyad/triad, and/or the broader family group. Likewise, a recent international survey found that music therapists working with families across various clinical settings tended to share and collaborate with families rather than act as musical experts [154]. However, similar to Epstein’s study [67], respondents remained flexible, adapting their approach to each individual context and assuming a more directive role when needed.

### 4.5. Strengths

A major strength of this integrative review was the research team’s clinical and research expertise in a range of health-related domains: nursing and midwifery, psychology, and MT. Additionally, each article was evaluated for quality via the MMAT, a validated appraisal tool. These factors minimised the risk of bias and prompted vigorous, insightful discussion during the review process whenever any issues arose. We employed a well-designed search strategy, systematically investigating literature across seven interdisciplinary databases, conducting electronic hand searches, and assessing grey literature. Adherence to strict inclusion and exclusion criteria ensured that only the most relevant studies were included. The use of MAXQDA software to document evolving themes and sub-themes via code creation, and a Creative Coding Map, assisted in the visualisation of themes and their connections to each other, and limited confirmation bias. This review also delivered contemporary findings, with most studies (*n* = 9) conducted in the past five years.

### 4.6. A Challenge

A key challenge was assessing studies in which MT commenced in the NICU and continued after hospital discharge, as the experience of MT in the NICU may have influenced post-discharge responses. However, we separated the results as far as practicable, primarily reporting post-discharge outcomes in the themes and discussion sections. If it was unclear to which phase a parent was referring, we omitted it from our data analysis.

### 4.7. Limitations

Limitations included restricting studies to publications in the English language, which may have contributed to skewed results and selection bias. However, despite initially considering articles in all languages, no additional relevant publications were found. Excluding grey literature, such as conference presentation abstracts and unpublished PhD theses, may also be considered a limitation. Nonetheless, some pertinent examples were mentioned in our Introduction. A further limitation might have been the absence of a theoretical framework to guide the IR process, as recommended by certain researchers [54,55]. Instead, similar to many IRs [155], we employed a methodological framework to direct and organise our findings.

The focus on certain populations and locations worldwide may also be perceived as a limitation. However, as many low-income nations have no access to MT, it was unavoidable. Some quantitative studies had insufficient preterm infant [44,45] and parent [59,60] participants for statistical analyses, with only trends reported. Therefore, results were interpreted cautiously due to the studies’ relatively small sample sizes. Lastly, data on the impact of MT post-discharge on fathers, older siblings, and caregivers from non-nuclear families were relatively scarce, although this may have been due to their absence in MT sessions and/or the gender focus of the included studies.

## 5. Implications

### 5.1. Implications for Research

Recommendations for future research emerge from this integrative review and relevant contemporary literature sources. Global advances in reproductive health and the increasing legal rights accorded to non-nuclear families in many countries [156,157,158] underscore the necessity for inclusive MT research after hospital discharge that incorporates preterm infants and their caregivers from varied kinship structures wherever possible, such as adoptive families, culturally and linguistically diverse populations, extended family, First Nations people, foster parents, intended (via surrogacy) parents, same-sex partners, single parents, and transgender or gender-diverse parents. This would complement MT clinical practice in the NICU [159,160,161,162,163,164], and post-discharge [38,165], where these populations are already represented.

Telehealth MT, which some post-discharge programs already use [38], has delivered promising results in other areas of paediatric [166,167,168] and adult MT clinical practice [169]. This warrants further exploration with families of preterm infants post-discharge, as some may be unable to participate in regular individual or group MT due to infant health concerns, parental work commitments, or living in remote areas. We also suggest developing and examining the effects of MT apps designed to encourage both paternal participation and overall parental self-efficacy, as this may support parents and other family members who are unable to participate in live MT sessions after their preterm infant is discharged.

Research is recommended into the effects of individual or group developmental MT sessions for long-term hospitalised preterm infants and their families in the NICU/PICU [170], as this is a relatively under-researched area of clinical practice. We also advocate more rigorous exploration of the effects of group-based MT sessions on infant development, parent/caregiver physical and mental health, and sibling wellbeing. There is additional scope for exploring the suitability of individual versus group MT for specific age groups and/or time frames post-NICU discharge. Examining the effects of MT post-discharge on distinct populations, such as extremely preterm, or low-birthweight infants and their families, is suggested. For instance, researchers could investigate the impact of MT follow-up clinics on preterm infants and children at high risk of developmental delays, as proposed by Pivovarnik [171]. This would depend on individual clinical contexts, as some hospitals and/or countries may not offer MT as part of post-NICU care.

Future research could also compare the effectiveness of individual post-discharge MT approaches and treatment methods (as identified in this integrative review) in relation to outcomes such as parent–infant bonding, infant neurodevelopment, and family mental health. In line with Ghetti et al. [66], we consider that mixed methods research may be an optimal approach for collecting, analysing, and interpreting data, as using both quantitative and qualitative methods would ensure a deeper understanding of preterm infants and their families’ reactions to—and experiences of—MT post-discharge and how this subsequently influences their lives [172,173].

### 5.2. Implications for Clinical Practice

In some countries, MT clinical practice with the families of preterm infants post-discharge precedes research. In Italy [40] and the USA [38], certain hospitals already provide follow-up MT for preterm infants and their families. For those countries where post-discharge MT is still evolving, we recommend focusing on the following areas:

1. Introducing face-to-face and/or telehealth MT programs in the first six months after hospital discharge;
2. Conducting community, hospital, or home-based MT programs (either individual or group sessions) from six months post NICU-discharge to preschool age;
3. Promoting collaboration between long-term follow up clinics and MT clinicians;
4. Offering online MT sessions for families in remote locations, or those unable to attend in person on a regular basis.

## 6. Conclusions

This review’s findings suggest that MT could play a significant role in creating supportive environments in which parents can develop skills, tools, and resources to build communication and connection between themselves and their preterm infants, as well as other family members. In addition, preterm infants and toddlers may increase their developmental skills via MT sessions post-discharge. There are, nevertheless, challenges that need to be addressed, such as caregivers’ emotional and physical health, parental reluctance to sing and/or make music, the lack of paternal or other family representation, and issues related to research implementation. Due to the limited research in this area, additional studies are required to ascertain the impact of MT on preterm infants and their parents/caregivers after hospital discharge. We recommend mixed method studies as a suitable methodology for this purpose.

## Figures and Tables

**Table 1:** Search terms and strategies.

Search No.	Search Terms and Strategies
Search 1.	preterm OR prematur* OR infant, premature
Search 2.	famil* OR parent*
Search 3.	“music therap*”
Search 4.	family-centred OR family-centered OR “family centred” OR “family centered”
Search 5.	intervention OR “early intervention”
Search 6.	post-discharge OR “post-discharge” OR “post-hospital discharge” OR “post hospital discharge” OR “after discharge” OR “after hospital discharge”
Search 7.	“developmental follow-up” OR “developmental follow up” OR follow-up OR “follow up”
Search 8.	“long-term development” OR “long term development” OR “longterm development”
Search 9.	Search 1 AND 2
Search 10.	Search 9 AND 3
Search 11.	Search 10 AND 4
Search 12.	Search 10 AND 5
Search 13.	Search 10 AND 6
Search 14.	Search 10 AND 7
Search 15.	Search 10 AND 8

**Table 2:** Occurrence of integrative review sub-themes.

	Creating Supportive Environments			Developing Skills, Tools and Resources			Acknowledging Challenges			Building Relationships		
author/s year	a physical or metaphorical space	a space for insight and self-expression	a space of calmness or engagement	infant development	parent development	music therapist development	infant challenges	parent challenges	research challenges	infants and parents	infants and others	parents and others
Standley et al. [40]			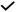	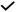			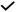		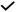	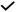	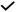	
Walworth [45]	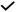		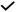	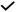	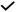				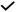	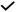	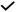	
Hamm et al. [46]			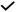	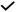					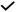	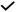		
Bieleninik et al. [67]	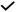	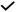	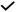	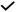	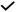	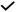			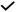	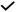	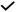	
Ghetti et al. [64]	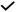		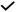	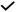	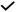	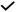	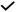	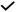	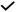	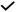	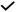	
Calderon-Noy and Gilboa [65]	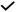	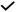	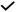	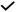	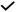		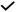	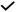	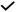	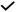	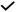	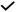
Epstein et al. [66]	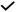	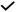	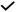	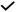	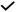	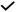	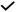	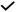	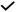	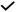	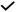	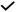
Howden et al. [67]	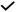	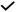			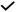			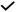	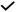	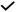	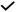	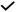
Gaden et al. [64]		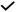	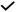		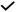	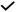		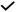	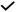	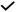		
Epstein et al. [65]	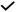	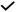	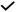	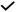	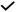	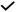		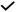	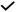	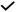	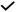	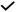
Ghetti et al. [66]				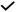	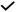			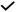	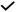	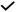		
Epstein [67]	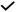	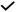	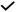	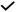	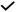	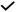	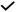	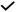	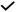	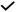	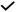	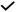

## Data Availability

The data from this review are available in the journal articles included in this review, which are available in the following databases: APA PsycInfo, CINAHL Fulltext, Ovid MEDLINE, ProQuest Central, SAGE Publishers, Scopus, and Web of Science.

## Supplementary Materials

The following supporting information can be downloaded at https://www.mdpi.com/article/10.3390/ijerph21081018/s1, Table S1—Characteristics of included studies; Additional Notes S1—Integrative themes: Word definitions. References [174,175,176,177,178,179,180,181,182,183,184,185,186,187,188,189,190] are cited in Supplementary Materials.
