# Supplementary material for: Music Therapy with Preterm Infants and Their Families after Hospital Discharge: An Integrative Review
Source: Int J Environ Res Public Health. 2024 Aug 2;21(8):1018. doi: 10.3390/ijerph21081018 (PMC11354888; doi:10.3390/ijerph21081018)
Supplement: Supplementary file 1 [file ijerph-21-01018-s001.zip › ijerph-3088274-supplementary.pdf]

Table S1. Characteristics of included studies

Author/s Year, Country	Study Aim/s	Participants Setting/s	Study Design	Interventions, Therapeutic Techniques	Data Collection and Analysis	Results/Findings	Quality Score
Standley et al. [44]<br><br>2009<br><br>USA	Investigate effects of structured group music sessions in toddler development	*Participants*<br>Toddlers (n=22) aged 12-24 months, equally divided into experimental (EG) and control groups (CG), with each comprising prematurely born toddlers at risk of developmental delay (EG, n=3, CG, n=2) and typically developing children (EG, n=8, CG, n=9)<br><br>*Setting*<br>Local hospital	Quantitative<br><br>Pilot Study<br><br>Experimental and Control Groups (Convenience Sampling)	Highly structured 25-minute (min) group music lessons twice a week x 12 weeks<br><br>Activities addressed communication, motor, parent-child, problem-solving, and social learning domains<br><br>Parents were instructed in activities, aims and objectives, with information sheets providing tips for home use	*Data Collection*<br>- Music group video recordings<br>- 29-item checklist adapted for study from:<br>Infant-Toddler Social and Emotional Assessment (ITSEA) and<br>Standley-Hughes Checklist (SHC)<br><br>*Data Analysis*<br>Trained observers viewed recorded sessions, using checklist to score individual children's behaviour after:<br>- EG – 4-7 music sessions<br>- CG – 1 music session<br>Mann Whitney U Test: compared differences (diffs.) in EG and CG group developmental skill scores<br>Paired *t*-test: determined diffs. between groups in music, cognitive, combined social/motor skills	- Significant (sig.) increase in developmental skill scores after participation in 4-7 music sessions<br>- Mean score for EG almost twice that of CG<br>- Music group demonstrated significantly higher music and cognitive skills compared to CG<br>- No sig. difference between EG and CG scores for social/motor skills<br>- No sig. correlation between participants' chronological age and developmental skills scores<br>- Sustained attention and initiating of responses during repeated activities indicated occurrence of infant learning across time<br>- Trend towards higher degree of developmental delay in music group's preterm toddlers but sample size (n=3) too small for analysis	**7/7**<br>S1. Yes<br>S2. Yes<br>4.1 Yes<br>4.2 Yes<br>4.3 Yes<br>4.4 Yes<br>4.5 Yes

Table S1. Characteristics of included studies (continued)

Author/s Year, Country	Study Aim/s	Participants Setting/s	Study Design	Interventions, Therapeutic Techniques	Data Collection and Analysis	Results/Findings	Quality Score
Walworth [45]<br><br>2009<br><br>USA	Investigate developmental responses (preterm and full term infants) and parental receptivity during toy play after MT intervention	*Participants*<br>Caregiver-infant dyads (n=56) equally divided into experimental and control groups of children born premature (EG, n=7, CG, n= not specified) or full term EG, n=21, CG, n=not specified), with caregivers self-selecting groups.<br>Children (7-24 months)<br>Caregivers (mothers, fathers, grandparents, nannies)<br><br>*Setting*<br>Location not specified (regional hospital, according to Walworth's PhD thesis, [1] (p. 31)	Quantitative<br><br>Quasi-experimental matched subjects with posttest only	EG – weekly 30-min developmental music groups, with minimum attendance of three groups in eight weeks before video recorded developmental assessment (parent-infant toy play)<br>Group musical activities encouraged infant social learning in environment where social behaviours were modelled by music therapists, caregivers, and other participants (infants/parents)<br><br>CG<br>- Developmental assessment only	*Data Collection*<br>- Music group video recordings<br>- Infants - Ages & Stages Questionnaire (ASQ)<br>- Barratt Simplified Measure of Social Status (BSMSS)<br>- Adults - Beck Depression Inventory (BDI)<br>- Parent Perception/Music Use questionnaire (PPQ) (self-developed by researcher)<br>*Data Analysis*<br>SCRIBE 4.0.4 software program for behavioural observation<br>*Inferential Statistics*<br>2-way Analysis of Variance (ANOVA) - Parents: diffs. between groups for: positive and negative parent behaviours.<br>Infants: diffs. in social and nonsocial infant behaviors between groups<br>Mann Whitney U Test<br>- analysed 4 groups of questionnaire responses<br>Paired *t*-test - diffs. in time spent in toy play between groups<br>Pearson correlation coefficient (PCC) for intraobserver reliability<br>Descriptive statistics<br>- EG preterm, EG and CG full term social/alone behaviours	ASQ - no significant pre-study differences between groups<br>BSMSS, BDI, PPQ - no significant differences between groups<br><br>Parent responsiveness:<br>- No significant differences but trend towards EG engaging in more positive interactions with infants during toy play than CG<br><br>EG preterm and term infants:<br>- significantly more social toy play with caregivers than control group ($p = < .05$)<br>- ages positively correlated with skills scores<br><br>CG preterm and term infants:<br>- ages negatively correlated with skills scores<br><br>EG preterm infants:<br>Although no. preterm infants too low for statistical analysis, descriptive findings suggest:<br>- no difference between EG preterm and EG matched age term infants in time spent engaging in social or alone behaviours<br>- more social and less alone behaviours in EG preterm infants than term matched age CG infants	**7/7**<br>S1. Yes<br>S2. Yes<br>3.1 Yes<br>3.2 Yes<br>3.3 Yes<br>3.4 Yes<br>3.5 Yes

Table S1. Characteristics of included studies (continued)

Author/s<br>Year,<br>Country	Study Aim/s	Participants<br>Setting/s	Study<br>Design	Interventions,<br>Therapeutic<br>Techniques	Data Collection<br>and<br>Analysis	Results/Findings	Quality<br>Score
Hamm et al. [46]<br><br>2017<br><br>USA	Measure changes in standardised developmental assessment scores at 6 and 12 months for preterm infants who participated in structured developmental MT sessions and compare with matched controls	*Participants*<br>Cases (n=10)<br>Former NICU patients who participated in weekly group MT sessions with caregivers<br><br>Controls (n=30)<br>Former NICU patients who did not attend MT sessions post-hospital discharge<br><br>*Setting*<br>Children's hospital	Quantitative<br><br>Case-Control Study<br><br>Cases and controls underwent retrospective comparison of developmental assessment scores	Weekly 45-min MT sessions using adapted Bright Start Cognitive Curriculum for Young Children,<br>a highly structured program involving developmental coaching via guided activities to encourage parent-infant interaction<br><br>Classes also welcomed typically developing children born at term	*Data Collection*<br>Developmental Assessment of Young Children (DAYC)<br><br>*Data Analysis*<br>SPSS, version 24 software<br>- Chi-square test compared:<br>Sex<br>White matter injury<br>Congenital Abnormality<br>Physical, Occupational & Speech Therapies prior to 6mths postmenstrual age<br>Maternal Education<br>- Two-tailed *t*-test compared:<br>Gestational age (GA) at birth	- No difference between cases and controls at baseline.<br>- After controlling for baseline DAYC scores, attending MT sessions was significantly associated with increases in 12-month scores in cognition, communication, and motor domains<br>- Post hoc analysis demonstrated cognitive and communication scores increased with higher MT attendance, however, motor scores did not increase.<br>- Inclusive developmental MT programs after hospital discharge may be beneficial for former preterm infants at high-risk of developmental delay, with specific objective of encouraging enhanced parent-infant communication	**7/7**<br>S1. Yes<br>S2. Yes<br>3.1 Yes<br>3.2 Yes<br>3.3 Yes<br>3.4 Yes<br>3.5 Yes

Table S1. Characteristics of included studies (continued)

Author/s<br>Year,<br>Country	Study Aim/s	Participants<br>Setting/s	Study<br>Design	Interventions,<br>Therapeutic<br>Techniques	Data Collection<br>and<br>Analysis	Results/Findings	Quality<br>Score
Bieleninik et al. [59]<br><br>2020<br><br>Poland	Evaluate:<br>1. feasibility of LongSTEP MT protocol procedures with Polish cohort before planned multi-site study implementation;<br>2. acceptability, integrability, and safety of MT intervention;<br>3. adaptability of outcome measures to Polish parents	*Participants*<br>*NICU*<br>Preterm infants born ≤ 35 weeks GA; medically stable<br>2 single babies<br>2 sets of twins<br>(n=6)<br>Mothers (n=4)<br><br>*Post-discharge*<br>Preterm infants at least 3 months corrected age (CA)<br>1 single baby<br>2 sets of twins<br>(n=6)<br>Older siblings (not specified)<br>Mothers (n=3)<br>Fathers (n=3)<br><br>*Setting/s*<br>NICU<br>Participant homes<br>Muzka MT center	Feasibility exploration at clinical and methodological levels<br><br>Explanatory/ Pragmatic	*NICU*<br>23 x 20-30 min MT sessions, with twice weekly MT for each family<br><br>*Post-discharge*<br>8 x 45-min MT sessions, with two per family across three-month-timespan post-discharge<br><br>Parent-infant musical interactions to encourage bonding and co-regulation, supported by music therapist	*Data Collection - Quantitative*<br>- Edinburgh Postnatal Depression Scale [EPDS]<br>- Generalized Anxiety Disorder Assessment [GAD7]<br><br>*Data Collection - Qualitative*<br>2 x semi-structured interviews (follow-up NICU and follow-up post-discharge)<br><br>*No Quantitative Data Analysis*<br>No statistical analysis due to small sample size, however, trends reported<br><br>*No Qualitative Data Analysis*<br>No qualitative data analysis, however semi-structured interviews provided citations of participant feedback	*Quantitative Results*<br>- EPDS – depression decreased (n=2)<br>and depression increased (n=10<br>- GAD7 – anxiety decreased (n=3)<br><br>*Qualitative Results*<br>- MT in NICU well-received by mothers<br>- MT post-discharge well-regarded and considered acceptable by parents<br><br>*Other Findings*<br>- No adverse effects in NICU or post-discharge resulting from MT participation<br><br>- MT appeared to contribute to a more positive experience of post-NICU discharge transition to home environment<br>- All planned MT sessions occurred, with only mothers attending NICU MT and fathers attending most sessions post-discharge<br>- Study confirmed feasibility of implementing – and evaluating – condensed LongSTEP protocol, thereby addressing previously unexplored area, (i.e., effective approaches for appraising multifaceted research procedures)<br>- MT intervention deemed appropriate for Polish context	**7/7**<br>*Qual*<br>S1. Yes<br>S2. Yes<br>1.1 Yes<br>1.2 Yes<br>1.3 Yes<br>1.4 Yes<br>1.5 Yes<br><br>**6/6**<br>*Quant*<br>S1. Yes<br>S2. Yes<br>4.1 Yes<br>4.2 Yes<br>4.3 Yes<br>4.4 Yes<br>4.5 N/A

Table S1. Characteristics of included studies (continued)

Author/s Year, Country	Study Aim/s	Participants Setting/s	Study Design	Interventions, Therapeutic Techniques	Data Collection and Analysis	Results/Findings	Quality Score
Ghetti et al. [60]<br><br>2021<br><br>Norway	Evaluate feasibility, acceptability, and suitability of elements of LongSTEP MT therapy protocol with Norwegian cohort before planned multi-site study implementation	*Participants*<br>Preterm infants: born ≤ 35 weeks GA; medically stable<br>- NICU (n=3)<br>- Post-discharge (n=2)<br><br>Primary caregivers:<br>Two MT sessions weekly in NICU and at least five of six sessions post-discharge<br><br>- NICU<br>Mothers (n= 3)<br>Fathers (n=3)<br>- Post-discharge<br>Mothers (n=2)<br>Fathers (n=2)<br>Older siblings (n=not specified)<br><br>*Setting/s*<br>NICU<br>Family home or hospital post-discharge	Quantitative and Qualitative<br><br>Critical Realist Perspective<br><br>Nonrandomised feasibility study	*NICU*<br>Twice weekly 45-50 min MT sessions, with 25-40 mins actively engaging with infant (4-7 sessions per family)<br><br>*Post-discharge*<br>Twice monthly 50-60 min sessions for three months (4-6 sessions per family)<br><br>Music therapist supported parent-infant musical, and nurturing, interactions to encourage mother-infant bonding, infant regulation and/or co-regulation	*Data Collection - Quantitative*<br>Validated Norwegian versions of:<br>- Postpartum Bonding Questionnaire (PBQ),<br>- Mother and Baby Interaction Scale (MABISC)<br>- Mother-to-Infant Bonding Scale (MIBS)<br>- (EPDS)<br>- GAD-7)<br>- Parental Stress Scale (PSS)<br>- Ages and Stages Questionnaire (ASQ: SE-2)<br><br>*Data Collection - Qualitative*<br>Audio-recorded 30-45 min semi-structured interviews with caregivers after last MT session<br><br>*Data Analysis – Quantitative*<br>Descriptive statistics<br><br>*Data Analysis – Qualitative*<br>Inductive thematic analysis	- In general, parents willingly engaged in MT research in which parental voice was primary instrument in NICU and after hospital discharge<br>- Parents found proposed protocol acceptable and suitable, with these variations to main study design requested:<br>1. assess fathers' mental health (GAD-7 and PSS)<br>2. feeding and sleeping questions removed from ASQ:SE-2 due to non-applicability at discharge or 3 months CA<br>3. include additional information re: MT aims and processes before commencing sessions<br><br>- PBQ retained due to psychometric robustness and extensive implementation in research, with MABISC and MIBS discarded<br><br>*Other Findings*<br>- Resources acquired in MT transferred to parent-infant interactions in daily life<br><br>- Via MT participation, parents acknowledged their voices' centrality in building and maintaining connections with their infants.	***7/7***<br>*Qual*<br>S1. Yes<br>S2. Yes<br>1.1 Yes<br>1.2 Yes<br>1.3 Yes<br>1.4 Yes<br>1.5 Yes<br><br>***6/6***<br>*Quant*<br>S1. Yes<br>S2. Yes<br>4.1 Yes<br>4.2 Yes<br>4.3 Yes<br>4.4 Yes<br>4.5 N/A

Table S1. Characteristics of included studies (continued)

Author/s Year, Country	Study Aim/s	Participants Setting/s	Study Design	Interventions, Therapeutic Techniques	Data Collection and Analysis	Results/Findings	Quality Score
Calderon-Noy & Gilboa [61]<br><br>2021<br><br>Israel	Explore novel MT method for encouraging communicative parental efficacy with music (CoPE with music), in mothers and infants after NICU discharge	*Participants*<br>Mother and preterm infant dyad<br><br>*Setting*<br>University MT clinic	Qualitative<br><br>Case Study	1x weekly 90-min MT session for eight weeks<br>Each session, dependent on dyad's immediate needs, involved:<br>1. Containment, where music therapist created space for mother to verbalise issues related to child's hospitalisation and/or life post-hospital discharge (if baby asleep or calm)<br>2. Modelling, where music therapist suggested/modelled musical interactions with baby<br>3. Practising, where mother practised what music therapist just modelled	*Data Collection*<br>Interviews prior to and after treatment<br><br>Audio recordings of MT sessions<br><br>*Data Analysis*<br>Audio recording transcription followed by content analysis of:<br>- Vocal interactions<br>- Music and movement improvisations	CoPE with music method supported mother in case study to acquire communicative parental efficacy via:<br>- verbally processing challenges experienced during her infant's preterm birth, NICU hospitalisation and family life after hospital discharge<br>- observing musical interactions between her infant and music therapist<br>- practicing musical and vocal communications with her baby during MT sessions<br>- transferring skills acquired in MT sessions to situations experienced in daily life	**7/7**<br>S1. Yes<br>S2. Yes<br>1.1 Yes<br>1.2 Yes<br>1.3 Yes<br>1.4 Yes<br>1.5 Yes

Table S1. Characteristics of included studies (continued)

Author/s Year, Country	Study Aim/s	Participants Setting/s	Study Design	Interventions, Therapeutic Techniques	Data Collection and Analysis	Results/Findings	Quality Score
Epstein et al. [62]<br><br>2022<br><br>Israel	Explore personal experience of Israeli parents actively engaging in MT with their preterm infants after hospital discharge	*Participants*<br>Families (n=5), included:<br>- male/female couples (n=2)<br>- mother in couple (n=2)<br>- Single mother (n=1)<br><br>Participants had not engaged in MT during hospitalisation<br><br>Preterm infants: born ≤ 35 weeks GA and between 6-9 months CA by end of MT sessions (n=5)<br><br>*Setting*<br>Family homes	Qualitative<br><br>Purposeful sampling process<br><br>Interpretative Phenomenological Analysis (IPA)	1 x monthly 50-60-min MT sessions for 6 to 7 months<br><br>Parent-infant musical interactions to encourage bonding and co-regulation, supported by music therapist	*Data Collection*<br>Two-Part Interviews, incorporating:<br>1. Semi-structured interview (SSI) exploring:<br>- participant experiences of MT after discharge<br>- barriers and facilitators to engaging in parent-infant musical dialogues<br>2. Adapted Interpersonal-Process Recall (IPR) procedure, where participants listen to – and comment on – self-selected audio MT session excerpts<br><br>*Data Analysis* – IPA Method<br>SSI data compared/ integrated with IPR data via:<br>- Individual interview transcriptions (SSI and IPR)<br>- Individual descriptive/ interpretive text processing<br>- Development of recurring themes within individual responses<br>- Overarching themes emerge across all participant responses	Two Overarching Themes<br>1. MT may potentially enhance parents' ability to: communicate with their infants, develop (inner) creative resources, and acquire a sense of musical/parental competence<br>2. Parental ability to engage in MT depends on meeting certain emotional and musical preconditions<br><br>Reflections<br>- Findings may not translate to other countries with: differing cultural/social contexts and/or other clinical MT and research practices<br>- Findings filtered through lens of first author's beliefs regarding research and own clinical experience	**7/7**<br>S1. Yes<br>S2. Yes<br>1.1 Yes<br>1.2 Yes<br>1.3 Yes<br>1.4 Yes<br>1.5 Yes

Table S1. Characteristics of included studies (continued)

Author/s Year, Country	Study Aim/s	Participants Setting/s	Study Design	Interventions, Therapeutic Techniques	Data Collection and Analysis	Results/Findings	Quality Score
Howden et al. [63]<br><br>2022<br><br>Australia	Explore two mothers' experiences of reflective lullaby creation after NICU discharge	*Participants*<br>Mothers (n=2)<br>Preterm Infants (n=2)<br>Older sibling (n=1)<br><br>*Setting/s*<br>3 face-to-face sessions at family home<br><br>1 online session on ZOOM	Qualitative<br><br>Pilot study<br><br>Descriptive phenomeno-logical approach	2-month period<br>4 x 60 min sessions<br><br>Parent-infant play songs, comprising singing and vocal interactions<br><br>Acoustic guitar accompaniment<br><br>Favourite songs and lullabies<br><br>Songwriting with parent	*Data Collection*<br>Semi-structured interviews<br><br>*Data Analysis*<br>Microanalysis in Music Therapy framework [2], being:<br>1. Transcribe interview<br>2. Identify main statements<br>3. Create structural meaning unit<br>4. Create experienced meaning units<br>5. Develop individual distilled essence<br>6. Identify Shared Themes	*Shared Themes*<br>1. Individual MT facilitated parent-infant engagement, self-reflection, and self-inquiry<br>2. Musical parent-infant interactions resulted in both mothers developing increased confidence in their musical abilities<br>3. Both mothers expressed satisfaction in creating song lyrics representing significant and memorable experiences with their baby in the NICU<br>4. Participating in MT resulted in both women reintegrating prior hobbies and internal resources into their current lives	**7/7**<br>S1. Yes<br>S2. Yes<br>1.1 Yes<br>1.2 Yes<br>1.3 Yes<br>1.4 Yes<br>1.5 Yes

Table S1. Characteristics of included studies (continued)

Author/s Year, Country	Study Aim/s	Participants Setting/s	Study Design	Interventions, Therapeutic Techniques	Data Collection and Analysis	Results/Findings	Quality Score
Gaden et al. [64]<br><br>2023<br><br>Argentina, Colombia, Israel, Norway, Poland	Investigate treatment fidelity (TF) in LongSTEP (LS) study by:<br>- assessing TF questionnaire reliability<br>- ascertaining uniformity of MT treatment delivery across international sites	*Participants*<br>135 families with infants born ≤ 35 weeks GA<br><br>10 music therapists<br><br>*Setting/s*<br>Seven NICUs in five countries<br><br>Family home, hospital, or other health care setting in five countries<br><br>Online (due to Covid-19 pandemic)	Quantitative<br><br>Assessing reliability and uniformity of MT treatment<br><br>Random TF participant selection via online software	*NICU*<br>3 x weekly 20-30 min sessions for minimum 2 weeks duration<br><br>*Post-discharge*<br>7 x 45-60 min sessions within 6 months<br><br>Parent-infant musical interactions to encourage bonding and co-regulation, supported by music therapist	*Data Collection*<br>NICU – audio recordings<br>Post-discharge – video recordings<br>*Data Analysis*<br>NICU - 72 sessions<br>Post-discharge - 40 sessions<br>Raters: 10 music therapists, 13 external raters<br>Tools:<br>- LS Treatment Delivery (TD) Tool, NICU and Post-discharge phase, external rater versions<br>- LS TD Tool D, NICU and post-discharge phase, music therapist self-rater versions<br>- LS Treatment Receipt (TR) Questionnaire (parents)<br><br>*Data Analysis*<br>Internal consistency<br>- Cronbach's alpha<br><br>Interrater reliability<br>- Intraclass correlation coefficient and Gwet's AC1<br><br>Categorical data analysis<br>- Frequency and percentage<br>Numerical data analysis<br>- Mean, standard deviation, range<br>Statistical Software<br>- R version 4.1.0	- Majority of TF questionnaires in both NICU and post-discharge phases demonstrated good internal consistency (*a* ≥ 0.70), with slightly lower score for NICU external rater questionnaire (*a* ≥ 0.66)<br>- All TF questionnaires demonstrated moderate interrater reliability, with NICU 0.43 (CI0.27, 0.58) and post-discharge 0.57 (CI 0.39, 0.73)<br>- Interrater reliability could be improved by additional rater training, such as viewing audiovisual recordings of sample MT sessions before rating process<br>- Consistency of MT treatment delivery deemed adequate across international sites as demonstrated by:<br>1. Mean TD composite score across raters = 4.88 (0.92) for NICU and 4.95 (1.05) post-discharge<br>2. Mean TD composite score across music therapists varied from 3.17 to 5.46 for NICU and 3.51 to 5.65 post-discharge<br>3. Mean TR parent scores very high for MT NICU only = 5.66 (0.50), MT post-discharge only = 5.65 (0.71) and MT both NICU and post-discharge 5.71 (0.40)<br>- Results suggest LongSTEP MT approach study highly applicable to diverse global clinical settings, with therapists implementing – and parents receiving MT according to LongSTEP protocol	**6/6**<br><br>S1. Yes<br>S2. Yes<br>2.1 Yes<br>2.2 Yes<br>2.3 Yes<br>2.4 N/A<br>2.5 Yes

Table S1. Characteristics of included studies (continued)

Author/s Year, Country	Study Aim/s	Participants Setting/s	Study Design	Interventions, Therapeutic Techniques	Data Collection and Analysis	Results/Findings	Quality Score
Epstein et al. [65]<br><br>2023<br><br>Israel	Address research gap by exploring Israeli parents' experiences of engaging in MT with their preterm infants in the NICU and after hospital discharge	*Participants*<br>Parents who had experienced LongSTEP MT in NICU and after hospital discharge (n=7)<br>Mothers (n=5)<br>Fathers (n=2)<br><br>*Setting/s*<br>NICU<br><br>Family homes	Interpretative Phenomenological Analysis (IPA)<br><br>Purposeful Sampling	*NICU*<br>3 x weekly 30-45 min MT sessions, ranging from 6 to 27 sessions<br><br>*Post-discharge*<br>6 to 7 months<br>1 x monthly 50-60 min MT sessions for 6 to 7 months<br><br>Parent-infant musical interactions to encourage bonding and co-regulation, supported by music therapist	*Data Collection*<br>Two-Part Interviews, involving:<br>1. SSI exploring:<br>- participant experiences of MT after discharge<br>- barriers/facilitators to engaging in parent-infant musical dialogues<br>2. Adapted IPR procedure, where participants listen to/comment on self-selected MT audio excerpts<br><br>*Data Analysis – IPA Method*<br>SSI data compared/integrated with IPR data via:<br>- Individual interview transcriptions (SSI and IPR)<br>- Individual descriptive/ interpretive text processing<br>- Development of recurring themes within individual responses.<br>- Overarching themes emerge across all participant responses	*Two Overarching Themes*<br>1. Music as therapeutic sanctuary<br>2. MT enables progressive incorporation of music into parent-infant relationship<br><br>*Other Findings*<br>- MT enabled parents to process NICU experience and access inner resources in hospital and afterwards<br>- For some parents, painful NICU memories may arise during post-discharge MT, with music therapists needing to maintain awareness and sensitivity if this occurs<br>- Findings may not translate to other countries with differing cultural/social contexts and/or other clinical MT and research practices	**7/7**<br>S1. Yes<br>S2. Yes<br>1.1 Yes<br>1.2 Yes<br>1.3 Yes<br>1.4 Yes<br>1.5 Yes

Table S1. Characteristics of included studies (continued)

Author/s Year, Country	Study Aim/s	Participants Setting/s	Study Design	Interventions, Therapeutic Techniques	Data Collection and Analysis	Results/Findings	Quality Score
Ghetti et al. [66]<br><br>2023<br><br>Argentina, Colombia, Israel, Norway, Poland	Investigate effect of MT on parent-infant bonding between caregivers and their prematurely born babies at 6 and 12 months corrected age	*Participants*<br>Families with infants born ≤ 35 weeks GA, and medically stable (n=213)<br><br>*Setting/s*<br>Seven NICUs in five countries<br><br>Family home, hospital, or other health care setting in five countries<br><br>Online (due to Covid-19 pandemic)	Quantitative Longitudinal<br><br>2 x 2 factorial, multinational, pragmatic randomised clinical trial	*NICU*<br>3 x weekly 20-30 min sessions<br><br>*Post-discharge*<br>6-month follow-up period post-hospital discharge<br>7 x 45-60 min sessions<br><br>Parent-infant musical interactions to encourage bonding and co-regulation, supported by music therapist	*Data Collection*<br>PBQ<br>EPDS<br>GAD-7<br>PSS<br>ASQ-3<br>ASQ:SE-2<br><br>*Data Analysis – Inferential Statistics*<br>Analysis of Covariance (ANCOVA)<br><br>*Data Analysis – Descriptive Statistics*<br>Group means measured by SD<br>Mean differences vs standard (95% confidence interval)<br><br>Statistical Analysis Software - R 4.1.0 (R Project for Statistical Computing)<br><br>Graphics<br>Matlab (Mathworks Inc)	Compared to standard care, no significant clinical effects of MT intervention on:<br>- mother-infant bonding (PBQ)<br>- maternal depression (EPDS)<br>- parental anxiety (GAD-7), (however MT may reduce anxiety in highly anxious fathers)<br>- parental stress (PSS)<br>- infant development (ASQ-3; ASQ:SE-2)<br><br>*Other Findings*<br>- No impaired bonding identified at baseline in either EG or CG<br>- No noted detrimental effects of intervention on parents or preterm infants<br>- Results generalisable to countries with high incidence of parental presence in NICU<br>- PBQ results differed from LongSTEP qualitative research, which suggested MT intervention promoted parent-infant communication and parental agency	**6/6**<br>S1. Yes<br>S2. Yes<br>2.1 Yes<br>2.2 Yes<br>2.3 Yes<br>2.4 N/A<br>2.5 Yes

Table S1. Characteristics of included studies (continued)

Author/s Year, Country	Study Aim/s	Participants Setting/s	Study Design	Interventions, Therapeutic Techniques	Data Collection and Analysis	Results/Findings	Quality Score
Epstein [67]<br><br>2023<br><br>Israel	Investigate construct of musical agency as conceptual framework for comprehending mechanisms involved in family-focused NICU and post-discharge MT.	*Participants*<br>Mother and preterm infant dyad<br><br>*Settings*<br>NICU<br><br>Family home	Qualitative<br><br>Case Study<br><br>Interpretative Phenomenological Analysis (IPA)	*NICU*<br>3 x weekly 30-45 min MT sessions for 19 days<br><br>*Post-discharge*<br>1 x monthly 50-60 min MT sessions for 6 months<br><br>Parent-infant musical interactions to encourage bonding and co-regulation, supported by music therapist	*Data Collection*<br>Agency and musical agency construct examined via social and psychotherapy theories<br><br>Case study MT session examples and interview collected for prior study via two-part interviews [61]<br><br>*Data Analysis*<br>Agency and musical agency constructs analysed within context of resource-oriented LongSTEP approach in general and specifically in relation to case study<br><br>MT session examples and interview analysed in prior study via IPA method [61] and adapted to case study format	- Resource-oriented foundation of LongSTEP approach aligns with musical agency framework, (i.e. parental voice is perceived as primary resource during MT), therefore parents are supported and empowered to actively engage with their infants via parent-led vocal interactions, subsequently mobilising their musical agency<br>- Case study confirmed abovementioned findings, with researcher-clinician nevertheless querying definition of providing support to parents in LongSTEP approach as mother in case study preferred music therapist lead MT sessions post-discharge	**7/7**<br>S1. Yes<br>S2. Yes<br>1.1 Yes<br>1.2 Yes<br>1.3 Yes<br>1.4 Yes<br>1.5 Yes

Table S1. Characteristics of included studies: (continued) Abbreviations

ANOVA	Analysis of Variance	LongSTEP	Longitudinal Study of music Therapy's Effectiveness for Premature infants and their caregivers
ANCOVA	Analysis of Covariance	MABISC	Mother and Baby Interaction Scale
ASQ-3	Ages & Stages Questionnaire®, Third Edition	MIBS	Mother-to-Infant Bonding Scale
ASQ:SE-2	Ages & Stages Questionnaires®: Social Emotional, Second Edition	MT	Music therapy
BDI	Beck Depression Inventory	NICU	Neonatal intensive care unit
BSMSS	Barratt Simplified Measure of Social Status	PBQ	Postpartum Bonding Questionnaire
CA	Corrected Age	PCC	Pearson correlation coefficient
CG	Control Group	PPQ	Parent Perception/Music Use
CoPE with music	Communicative Parental Efficacy with Music	PSS	Perceived Stress Scale
DAYC	Developmental Assessment of Young Children	RCT	Randomized controlled trial
EPDS	Edinburgh Postnatal Depression Scale	SD	Standard deviation
EG	Experimental Group	SHC	Standley-Hughes Checklist
GA	Gestational Age	SPSS®	Statistical Package and Service Solutions
GAD-7	Generalized Anxiety Disorder Assessment - 7	SSI	Semi-structured interview
ICC	Intraclass correlation coefficient	TD	Treatment delivery
IPA	Interpretative Phenomenological Analysis	TF	Treatment fidelity
IPR	Interpersonal-Process Recall	TR	Treatment receipt
ITSEA	Infant-Toddler Social and Emotional Assessment		

Additional Notes: S1 – Integrative review themes: Word definitions

Central Theme: Offering Opportunities for Development and Change via Music Therapy
The term 'offering' can be defined as willing to give somebody something [3], 'opportunity' denotes a time when something is possible to achieve or do [4], 'development' signifies undergoing a metaphoric process of growth [5], while 'change' means the process or outcome of transformation [6, 7]. 'Music therapy' can be defined as an evidence-based, allied health approach/profession in which university-trained music therapists support people across the lifespan to address their physical and/or mental health [8].

Theme 1: Creating Supportive Environments – in this context, 'creating' signifies enabling something to occur or exist [9], 'supportive' is providing encouragement or assistance [10], while the term 'environment' signifies the context, conditions or objects comprising a person's immediate surroundings [11].

Theme 2: Developing Skills, Tools, and Resources – 'Developing' can be defined as creating or producing something [12]. The term 'skill' may be defined as an acquired ability to do something well [13], whereas the term 'tool' is a therapeutic technique helping a person to achieve something [14]. 'Resource' is either an inner or outer source of strength to promote health and wellbeing [15].

Theme 3: Acknowledging Challenges – the term 'acknowledging' is defined as taking notice – or becoming aware – of something [16], whereas the term 'challenge' signifies a problem or task that tests an individual's ability to do something [17].

Theme 4: Building Relationships – in this context 'building' is defined as making something by assembling diverse components [18], whereas 'relationship' signifies an ongoing connection between people [19].

References – Supplementary Material

1. Walworth, D. D. The Effect of Developmental Music Groups for Parents and Premature or Typical Infants Under Two Years on Parental Responsiveness and Infant Social Development. Ph.D. Dissertation, Florida State University, FL, USA, 2007. http://purl.flvc.org/fsu/fd/FSU_migr_etd-1271
2. McFerran, K., & Grocke, D. Understanding Music Therapy Experiences Through Interviewing: A Phenomenological Microanalysis. In *Microanalysis in Music Therapy: Methods, Techniques and Applications for Clinicians, Researchers, Educators, and Students;* Wosch, T., & Wigram, T., Eds.; Jessica Kingsley: London, UK, 2007; pp. 273-284.
3. Oxford University. *Offer.* Oxford Advanced Learner's Dictionary. https://www.oxfordlearnersdictionaries.com/definition/english/offer_1?q=offer (accessed on 15 August 2023).
4. Oxford University. *Opportunity.* Oxford Advanced Learner's Dictionary. https://www.oxfordlearnersdictionaries.com/definition/english/opportunity_1?q=opportunity (accessed on 15 August 2023).
5. Merriam-Webster. *Development.* https://www.merriam-webster.com/dictionary/development (accessed on 15 August 2023).
6. Merriam-Webster. *Change.* https://www.merriam-webster.com/dictionary/change (accessed on 15 August 2023).
7. Oxford University. *Change.* Oxford Advanced Learner's Dictionary. https://www.oxfordlearnersdictionaries.com/definition/english/change_2 (accessed on 15 August 2023).
8. Australian Music Therapy Association. *What is Music Therapy?* Available online: https://www.austmta.org.au/about-us/what-is-mt/ (accessed on 10 September 2023).
9. Oxford University. *Create.* Oxford Advanced Learner's Dictionary. https://www.oxfordlearnersdictionaries.com/definition/english/create?q=create (accessed on 15 August 2023).
10. Oxford University. *Supportive.* Oxford Advanced Learner's Dictionary. https://www.oxfordlearnersdictionaries.com/definition/english/supportive?q=supportive (accessed on 15 August 2023).
11. Merriam-Webster. *Environment.* https://www.merriam-webster.com/dictionary/environment (accessed on 15 August 2023).
12. Merriam-Webster. *Develop.* https://www.merriam-webster.com/dictionary/develop (accessed on 15 August 2023).
13. Merriam-Webster. *Skill*. Merriam-Webster Online Dictionary. https://www.merriam-webster.com/dictionary/skill. (accessed on 15 August 2023).
14. Oxford University. *Tool.* Oxford Advanced Learner's Dictionary. https://www.oxfordlearnersdictionaries.com/definition/english/tool_1 (accessed on 15 August 2023).
15. Prüfer, F.; Joos, S.; Miksch, A. What Do Resource-Oriented Approaches Mean to General Practitioners and How Can They Be Facilitated in Primary Care? A Qualitative Study. *Evidence-based Complement. Altern. Med.* **2013**, *2013*. https://doi.org/10.1155/2013/187641
16. Merriam-Webster. *Acknowledge.* Merriam-Webster Online Dictionary. https://www.merriam-webster.com/dictionary/acknowledge (accessed on 15 August 2023).
17. OxfordUniversity. *Challenge.* Oxford Advanced Learner's Dictionary. https://www.oxfordlearnersdictionaries.com/deinition/english/challenge_1?q=challenge (accessed on 15 August 2023).
18. Oxford University. *Build.* Oxford Advanced Learner's Dictionary. https://www.oxfordlearnersdictionaries.com/definition/english/build_1?q=build (accessed on 15 August 2023).
19. American Psychological Association. *Relationship.* APA Dictionary of Psychology. https://dictionary.apa.org/relationship (accessed on 15 August 2023).
